# In Vitro Doxorubicin Delivery Using TPP–Folate-Dendrimer-Functionalized Gold Nanoclusters

**DOI:** 10.3390/ph19040572

**Published:** 2026-04-02

**Authors:** Mkhuseli Zenze, Moganavelli Singh

**Affiliations:** Nano-Gene and Drug Delivery Group, Discipline of Biochemistry, University of KwaZulu-Natal, Private Bag X54001, Durban 4001, South Africa; 213515339@stu.ukzn.ac.za

**Keywords:** PAMAM dendrimers, gold nanoclusters, folic acid, triphenylphosphonium cation, doxorubicin, delivery, cytotoxicity, mitochondria

## Abstract

**Background**: Cancer is a major health concern that significantly impacts the global population. Selective chemotherapeutic delivery is needed to improve the efficacy of cancer therapy while minimizing side effects in healthy cells. This study investigated the potential of gold nanoclusters (AuNCs) functionalized with poly(amidoamine) dendrimers (PAMAM) and folic acid (FA) to selectively deliver doxorubicin (DOX) to cancer cells that express the folate receptor (FR). **Methods**: AuNC synthesis was confirmed via UV–visible and Fourier transform infrared spectroscopy, nanoparticle tracking analysis, and transmission electron microscopy. Folic acid (FA) was incorporated for cell surface receptor targeting, while the triphenylphosphonium cation (TPP^+^) was added to improve mitochondrial localization. Cytotoxicity (MTT), apoptosis, caspase 3/7, mitopotential, and oxidative stress assays were assessed using human MCF-7 (breast adenocarcinoma), HeLa (cervical carcinoma), Caco-2 (colon adenocarcinoma), MDA-MB-231 (epithelial breast cancer), and the embryonic kidney (HEK293) cells. **Results**: Favorable DOX loading (>78%), with more than 90% of the drug released at pH 4.5, was achieved. A dose-dependent increase in cytotoxicity was observed, with IC50 values lower in cancer cells than HEK293 cells, indicating selective toxicity and minimal off-target effects. Targeting nanocomplexes produced the best responses in the mitopotential, caspase, and oxidative stress assays in HeLa and MCF-7 cells. **Conclusions**: The improved cytotoxicity in cancer cells may be due to folate-receptor-mediated cellular uptake, as well as the mitochondrial uptake of TPP^+^ nanocomplexes. This highlighted the potential of the drug–AuNC nanocomplexes to limit systemic side effects, proposing a potential novel strategy for drug delivery to cancer cells.

## 1. Introduction

Cancers, especially breast cancer, a growing worldwide concern, are characterized by the uncontrolled growth of abnormal cells in the breast tissue, and it is a complex and multifaceted condition [1]. It is the most commonly diagnosed cancer in women, but it affects both genders [2]. This disease has no geographical boundaries, affecting individuals from diverse backgrounds and regions [3]. According to the World Health Organization (WHO), breast cancer is the most frequently diagnosed cancer in women, with an estimated 2.4 million new cases reported in 2024 [4]. In 2022, there were an estimated 670,000 fatalities due to breast cancer, with an annual increase of 2.5% [5]. This highlights the widespread nature of the disease and underscores the urgent need for early detection and improved treatment strategies to prevent and manage it effectively.

Despite advancements in cancer treatment, breast cancer remains a significant challenge for modern medicine. Traditional chemotherapy has made considerable strides, but its non-specificity often leads to severe side effects and limited efficacy [6,7]. Nanomedicine has emerged as a promising field in the fight against cancer, offering innovative solutions to enhance the precision and effectiveness of cancer therapeutics [8]. The use of gold nanoclusters (AuNCs) to deliver doxorubicin (DOX), a potent chemotherapeutic agent, into cancer cells’ mitochondria has attracted considerable attention [9].

Mitochondria, commonly referred to as the “powerhouses” of the cell, play a crucial role in cellular energy production and the regulation of apoptosis, a process that involves the programmed death of cells [10]. Dysfunctional mitochondria are characteristic of many cancer types and enable their survival and proliferation [11]. To exploit this weakness, researchers have developed nanoparticles (NPs) that selectively target and deliver anticancer drugs, such as DOX, to mitochondria [12]. This precise delivery of the drug offers the potential to enhance its therapeutic effect while limiting harm to healthy tissues, thereby alleviating any severe side effects typically associated with conventional chemotherapy [13].

AuNCs are a unique group of nanomaterials composed of a small number of gold atoms (2 to about 100) arranged in a precise cluster structure [14]. These clusters exhibit unique optical, electronic, and catalytic properties due to their size-dependent quantum effects, making them ideal candidates in nanomedicine. Their small size, high surface area-to-volume ratio, and biocompatibility make them excellent carriers for a broad range of therapeutic agents [15,16]. Additionally, they can be functionalized with various targeting molecules, enabling them to navigate the complex environment of cancer cells with high precision [17]. Their application in cancer treatment lies in their capacity to transport therapeutic agents directly to tumor sites, thereby sparing healthy tissues from the harmful effects of chemotherapy [18,19].

Poly(amidoamine) (PAMAM) dendrimers have emerged as versatile and promising agents for modifying NPs, to enable the tailored delivery of drugs or genes to cells [20,21]. This synergistic combination of dendrimers and NPs represents a cutting-edge approach to revolutionize cancer therapy [22,23]. PAMAM dendrimers, with their well-defined structure and multiple functional groups, offer an ideal platform for engineering NPs as delivery systems. When strategically integrated into NPs, they enable the encapsulation, protection, and controlled release of therapeutic agents into the cancer cells [24]. The surface functionalities of dendrimers can be precisely tailored to enhance cellular targeting, improve drug uptake, and minimize off-target effects. The use of PAMAM dendrimers as functionalizing agents has the potential to increase drug bioavailability, enhance therapeutic efficacy, and mitigate the often-debilitating side effects associated with traditional chemotherapy [22,25].

Folic acid (FA)-mediated targeted drug delivery can selectively deliver therapeutic agents to tumor cells, thereby improving treatment outcomes and circumventing multidrug resistance [26]. The combination of FA-targeted therapy with other treatment modalities, such as chemotherapy, immunotherapy, or photodynamic therapy, presents a multifaceted approach to combating breast cancer progression and metastasis [27]. Despite these benefits in breast cancer therapy, several challenges need to be addressed. These include optimizing the design of FA-conjugated NPs to enhance tumor penetration, improving drug-loading efficiency, and minimizing premature drug release. The diverse expression of folate receptors (FRs) in breast cancer subtypes underscores the need for personalized treatment strategies tailored to individual patient profiles [28].

In cancer therapy, TPP^+^-conjugated chemotherapeutic agents have been shown to exhibit increased cytotoxicity in cancer cells due to their selective accumulation in cancer cell mitochondria [29]. Similarly, the TPP^+^-mediated delivery of antioxidants and anti-apoptotic agents demonstrates promise for mitigating mitochondrial dysfunction associated with neurodegenerative diseases and ischemia–reperfusion injury. Challenges such as optimizing the design of TPP^+^-conjugated compounds to enhance mitochondrial selectivity, improve linker stability, and minimize off-target effects must be addressed [30].

This preliminary study aims to investigate the delivery of DOX to selected breast cancer cells using TPP^+^-FA-PAMAM-modified AuNCs. FA is used to target the overexpressed FRs in breast cancer cells, and TPP+ is a potential mitochondria-targeting ligand. This research is unique and novel in that it proposes a nano-delivery formulation with potential dual targeting ability for enhanced drug delivery efficiency to breast cancer cells. Although other systems conjugated to TPP^+^ and PAMAM have been utilized, AuNCs have yet to be exploited for this purpose.

## 2. Results

### 2.1. AuNC Synthesis and DOX Encapsulation

AuNCs were successfully synthesized and functionalized with PAMAM, FA, TPP^+^, and PEG. A total of nine nanoparticles (TPP^+^ conjugated to the AuNCs would be henceforth abbreviated simply as TPP within the nanoconjugate) were formulated, viz., AuNC, PAM-AuNC, FA-PAM-AuNC, TPP-PAM-AuNC, TPP-FA-PAM-AuNC, PEG-PAM-AuNC, FA-PEG-PAM-AuNC, TPP-PEG-PAM-AuNC, and TPP-FA-PEG-PAM-AuNC. These functionalized formulations will be collectively referred to as FAuNCs. The AuNCs were functionalized with the cationic TPP^+^ moiety to improve mitochondrial localization upon cellular uptake. The TPP^+^ -targeted AuNCs (TPP-PAM-AuNC and TPP-FA-AuNC) were formulated by linking TPP^+^ to PAM’s surface amino groups to form TPP-PAM, which was then coupled with AuNCs to yield TPP-PAM-AuNCs. A similar approach was employed to conjugate FA-TPP-PAM and its PEGylated counterparts.

**Figure 1:**
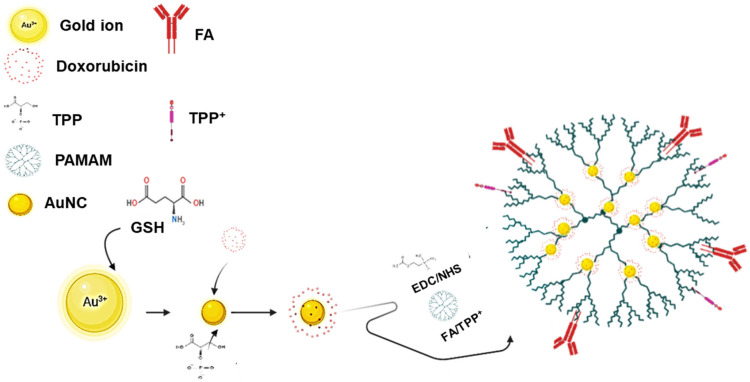
Illustration depicting the formulation of functionalized AuNC-DOX nanocomplexes. Created in BioRender. Mokhosi, S. (2026) https://BioRender.com/pw5i1t9 (accessed 3 December 2025).

DOX was encapsulated into the AuNCs during synthesis, followed by polymer functionalization. The drug was encapsulated within the ionically assembled AuNC–STPP complex via co-precipitation with sodium tripolyphosphate (STPP), which facilitated electrostatic interactions between the drug and the nanocluster matrix [31]. The AuNC had an EE of approximately 89.5%, which was the highest, and upon PAMAM modification, the EE decreased, where PAM-AuNCs had an encapsulation efficiency (EE) of around 85.4%, whereas FA-TPP-PAM-AuNCs had an EE of approximately 81%. The lowest EE recorded was 78.4% for TPP-PEG-PAM-AuNCs. Nonetheless, all synthesized FAuNCs had a desirable EE of more than 78%, suggesting potential for drug delivery. Figure 1 illustrates the overall formulation of the FAuNC-DOX nanocomplexes, and Table 1 lists the EEs of the different FAuNCs.

### 2.2. UV-Vis and FTIR Spectroscopy

**Figure 2:**
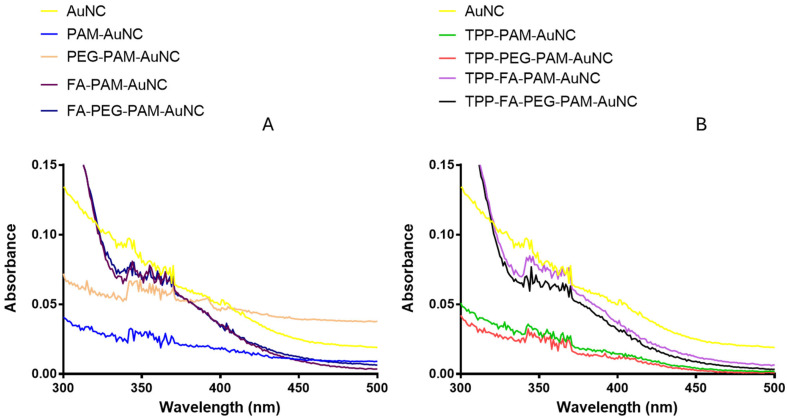
UV–vis spectra of (**A**) FAuNC without TPP^+^ and (**B**) TPP^+^-linked FAuNC.

The UV-vis spectroscopy (Figure 2) demonstrated the absorbance characteristics for the AuNC formulations between 200 and 800 nm. The unmodified AuNC exhibited absorbance peaks in the 300–400 nm range, which can be attributed to π-π* transitions characteristic of luminescent AuNCs and are consistent with the literature [32]. The absorbance decreases above 400 nm, suggesting that the visible spectrum is not very active.

There were noticeable changes in the absorbance profiles of the functionalized AuNCs (FAuNCs), viz., PAM-AuNC, PEG-PAM-AuNC, FA-PAM-AuNC, FA-PEG-PAM-AuNC, TPP-PAM-AuNC, TPP-PEG-PAM-AuNC, TPP-FA-PAM-AuNC, and TPP-FA-PEG-PAM-AuNC. The FAuNCs showed a blueshift relative to the unmodified AuNCs, with a λ_max_ in the 250–300 nm range. This suggested that polymer conjugation, which alters the electronic transitions of the AuNCs, is due to substantial changes in the surface chemistry.

**Figure 3:**
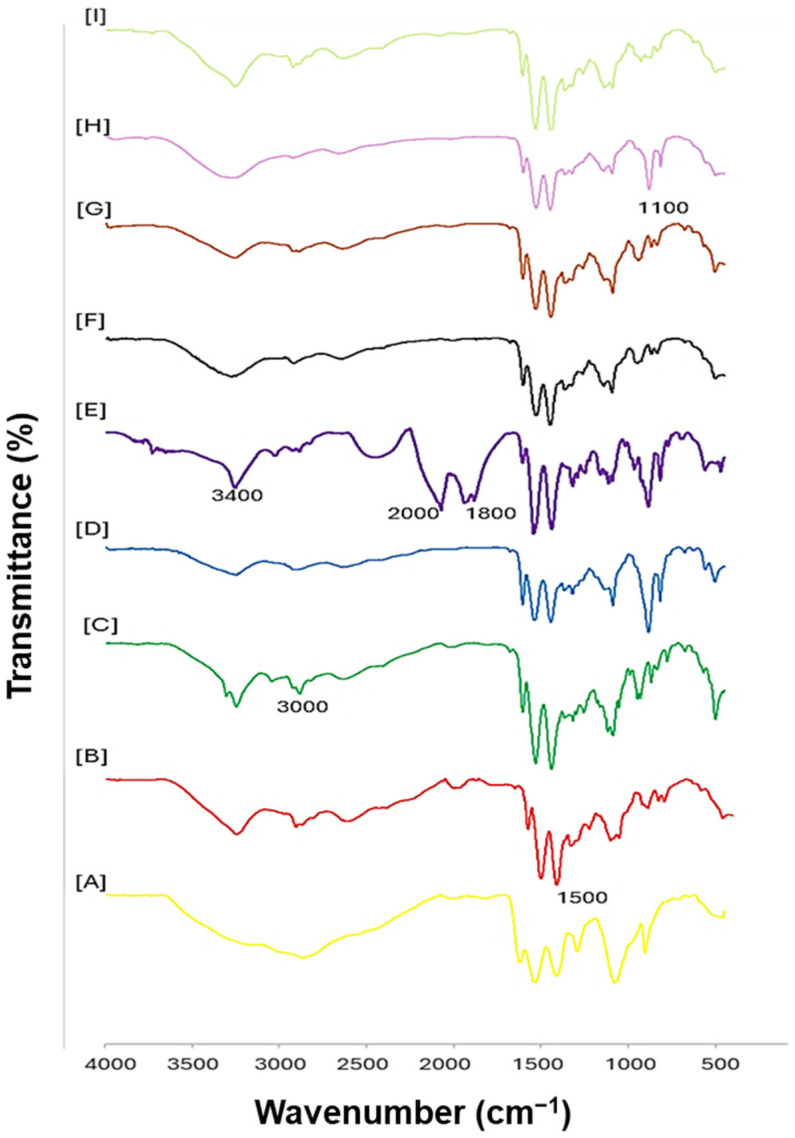
FTIR spectra of AuNCs and FAuNCs showing changes in bond vibrations of respective functional groups in the (**A**) AuNC, (**B**) PAM-AuNC, (**C**) PEG-PAM-AuNC, (**D**) FA-PAM-AuNC, (**E**) FA-PEG-PAM-AuNC, (**F**) TPP-PAM-AuNC, (**G**) TPP-PEG-PAM-AuNC, (**H**) TPP-FA-PAM-AuNC, and (**I**) TPP-FA-PEG-PAM-AuNC.

The FTIR spectra (Figure 3) show large peaks above 3000 cm^−1^, especially around 3500–3200 cm^−1^, suggesting the stretching vibrations of O-H or N-H. The O-H stretching in alcohols or carboxylic acids is characterized by a broad and intense peak for the FA-PEG-PAM-AuNC at 3400 cm^−1^. Additionally, the persistence of this broad band, along with slight broadening compared with PEG-PAM-AuNCs, suggests hydrogen-bonding interactions associated with the folic acid conjugation. The medium-intensity peaks between 2900 and 2800 cm^−1^ were probably caused by aliphatic hydrocarbons that correspond to C-H stretching vibrations. These peaks appear consistently throughout the spectra, indicating that the samples contained methyl/methylene groups or alkyl chains. In PEG-PAM-AuNCs, the relative increase in intensity in this region is consistent with the incorporation of PEG chains, which are rich in –CH_2_– groups. As the spectrum moves into the fingerprint area (1500–500 cm^−1^), it shows several distinct and strong peaks that are characteristic of aromatic C-H bending and C=C stretching vibrations [33]. In particular, FA-containing samples show enhanced aromatic signals in this region, consistent with the pteridine and benzoyl ring structures of folic acid, supporting successful ligand incorporation.

For TPP-PAM-AuNCs, peaks near 1700 cm^−1^ indicated the presence of carbonyl (C=O) groups from carboxylic acids, ketones, or aldehydes. In FA-functionalized samples, this region also shows a slight shift to ~1650 cm^−1^ together with increased intensity, which is consistent with amide bond formation following FA coupling to PAMAM’s surface amines. The peaks in PEG-PAM-AuNCs between 1600 and 1500 cm^−1^ are compatible with C=C stretching in alkenes or aromatic rings. This region’s fluctuations in peak sharpness and intensity highlight variations in molecular environments, including conjugation or substitution patterns. Specifically, the sharpening of peaks around ~1600 cm^−1^ in FA-PAM-AuNCs compared with PAM-AuNCs suggests the contribution of aromatic C=C vibrations from folate structures. The amide I band may be responsible for the sharp peak in the FA-PAM-AuNC at 1650 cm^−1^, but the peaks close to 1450 cm^−1^ may be the result of C-H bending vibrations in methyl or methylene groups. Importantly, the emergence of the amide I band at ~1650 cm^−1^ together with a weaker amide II feature around ~1540 cm^−1^ provides diagnostic evidence for amide linkage formation during ligand conjugation. The spectra also display peaks below 1000 cm^−1^, which are linked to out-of-plane deformations in aromatic compounds and C-C stretching or bending vibrations. Additional bands in the ~1100–1050 cm^−1^ region in PEG-modified samples are consistent with the C–O–C stretching of PEG ether linkages, further confirming PEG incorporation. A combination of hydrogen-bonded functional groups and aromatic or conjugated structures is suggested by the existence of these peaks, as well as the more general features at higher wavenumbers. The presence of aliphatic groups is confirmed by the constancy of C-H stretching vibrations across the spectra, and the molecular structures’ complexity is highlighted by the unique fingerprint area peaks, caused by the various functional groups or side chains [34,35].

### 2.3. Transmission Electron Microscopy (TEM) and Nanoparticle Tracking Analysis (NTA)

**Figure 4:**
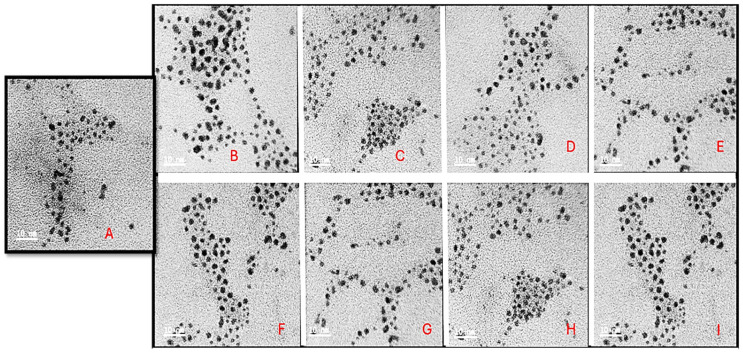
TEM micrographs of the (**A**) AuNC, (**B**) PAM-AuNC, (**C**) PEG-PAM-AuNC, (**D**) FA-PAM-AuNC, (**E**) FA-PEG-PAM-AuNC, (**F**) TPP-PAM-AuNC, (**G**) TPP-PEG-PAM-AuNC, (**H**) TPP-FA-PAM-AuNC, and (**I**) TPP-FA-PEG-PAM-AuNC. Scale bar = 10 nm.

**Figure 5:**
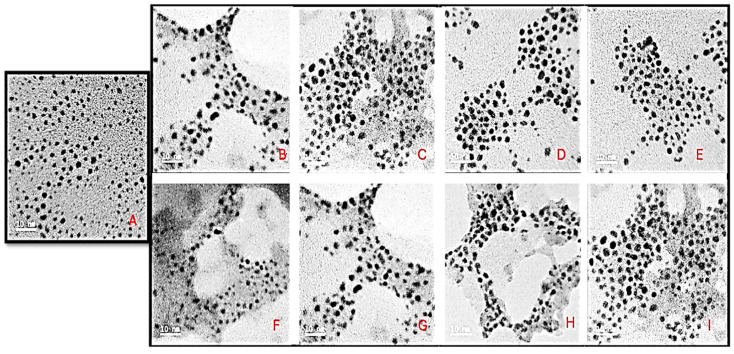
TEM micrographs of the (**A**) AuNC-DOX, (**B**) PAM-AuNC-DOX, (**C**) PEG-PAM-AuNC-DOX, (**D**) FA-PAM-AuNC-DOX, (**E**) FA-PEG-PAM-AuNC-DOX, (**F**) TPP-PAM-AuNC-DOX, (**G**) TPP-PEG-PAM-AuNC-DOX, (**H**) TPP-FA-PAM-AuNC-DOX, and (**I**) TPP-FA-PEG-PAM-AuNC-DOX. Scale bar = 10 nm.

TEM characterization revealed monodispersed circular AuNCs with an average diameter of 5 nm (Figure 4). Upon functionalization, the FAuNCs assumed a clustered appearance. This can be attributed to the PAMAM pocket spaces within the dendrimer, which enable AuNCs to fit within them and cluster to form a single nanocomplex. The FAuNCs had an average diameter of 10 nm but were not monodispersed, unlike the AuNCs. The drug-encapsulated nanocomplexes were relatively circular, with an average diameter of 12 nm (Figure 5), which was not significantly different from that of their counterparts without the drug. The non-significant increase in nanocomplex size can be attributed to the principle of TEM, as samples are analyzed in their dry state.

The Stokes diameter, or hydrodynamic diameter, accounts for the hydration layer surrounding NPs and is influenced by the diameter of the NP’s corona. Table 2 reflects the TEM and NTA (hydrodynamic) sizes of all FAuNCs and their nanocomplexes. The size distribution profiles from NTA are provided in Supplementary Figure S1. The AuNCs exhibited a negative zeta potential (−38.8 ± 0.5 mV) due to the presence of the anionic capping ligand glutathione, which stabilized the cluster. Upon PAMAM functionalization, all nanoclusters exhibited positive zeta potentials due to PAMAM’s high number of amine groups, which neutralized the negative charges due to the glutathione capping. Subsequent modifications reduced the hydrodynamic diameter to 110 nm, 100 nm, and 97 nm, respectively, upon functionalization with FA, TPP^+^, and TPP-FA. PEGylation resulted in a slight increase in the hydrodynamic size and was within accepted zeta-potential values.

FA functionalization of PAM-AuNCs resulted in minimal changes, with the FAuNC maintaining a relatively positive zeta measurement. Further functionalization with TPP^+^ (0.226 mM) enhanced the positive surface charge, yielding favorable high zeta potentials of 38.0 mV for FA-PAM-AuNCs and 43.4 mV for TPP-FA-PAM-AuNCs. These zeta potentials suggested that all FAuNCs were stable as they all exhibited zeta potentials > 20 mV.

### 2.4. Drug Release Profile

**Figure 6:**
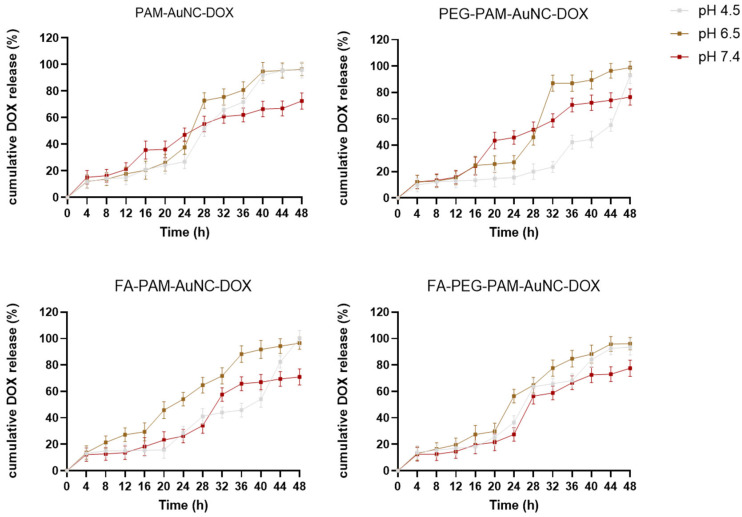
In vitro release profile of DOX from the FAuNC-DOX nanocomplexes at pH 4.5, 6.5, and 7.4. Data are represented as the mean ± SD *(n* = 3). Readings were taken at 4 h intervals over 48 h.

**Figure 7:**
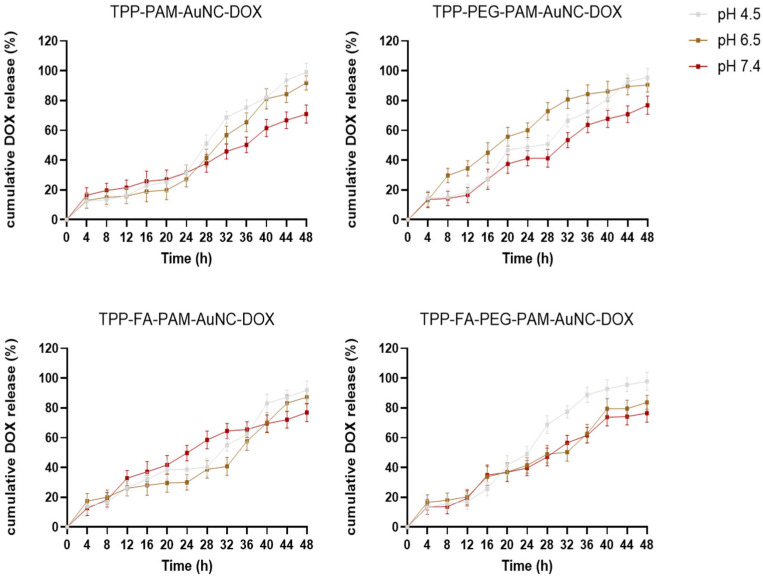
In vitro release profile of DOX from the TPP^+^ containing FAuNC-DOX nanocomplexes at pH 4.5, 6.5, and 7.4. Data are represented as mean ± SD *(n* = 3). Readings were taken at 4 h intervals over 48 h.

Figure 6 and Figure 7 reflect the drug release profiles for the different FAuNC formulations. PAMAM-AuNC-DOX showed slow release at pH 4.5, peaking at 95.88% after 48 h, and at pH 7.4, the release reached a plateau at 72.59%. The FA-PAMAM-AuNC-DOX demonstrated rapid release kinetics at pH 4.5, reaching 100% release in 48 h, but only 70.94% at pH 7.4. Every formulation showed a biphasic release pattern, with an initial slow phase. TPP-PAMAM-AuNC-DOX released 12.35% at pH 4.5 after 4 h with an accelerated release (99.06%) at 48 h. The TPP-PEG-FA-PAMAM-AuNC-DOX released 97.88% at pH 4.5 but only 76.59% at pH 7.4, with pH 4.5–6.5 promoting faster and more complete release. The prolonged release from PEG-PAMAM-AuNC-DOX was noteworthy. The cumulative release at pH 4.5 increased from 9.88% (4 h) to 92.94% (48 h), indicating greater drug–carrier interactions. Under acidic conditions, the initial burst release can be ascribed to surface-bound drug dissolution, which was particularly pronounced for PEG-FA-PAMAM-AuNC-DOX, with 13.88% release within 4 h. The cumulative drug release at acidic pH exceeded that at physiological pH, demonstrating the pH-responsive behavior of the FAuNPs. TPP-FA-PAMAM-AuNC-DOX, for example, released 92% of DOX at pH 4.5 compared to 76.94% at pH 7.4.

Five kinetic models were used to analyze the release kinetics of DOX from the different FAuNC nanocomplexes (PAM-AuNC, PAM-AuNC-DOX, PEG-PAM-AuNC-DOX, FA-PAM-AuNC-DOX, PEG-FA-PAM-AuNC-DOX, and their TPP^+^-conjugated equivalents) at pH 4.5, 6.5, and 7.4. The first-order (R^2^ = 0.958–0.988) and Korsmeyer–Peppas (R^2^ = 0.961–0.990) models consistently showed the strongest correlations for all nanocomplexes and pH levels, with diffusion and erosion being predominant. To further elucidate the release mechanisms, the Korsmeyer–Peppas release exponent (*n*) was calculated from the initial 60% of the release data (Table 3). At pH 7.4, *n* values ranged from 0.37 to 0.42, indicating Fickian diffusion-controlled release. However, at pH 4.5, *n* values increased to 0.44–0.58, suggesting anomalous (non-Fickian) transport where drug release is governed by combined diffusion and polymer relaxation/swelling. This pH-responsive behavior is particularly advantageous for tumor-targeted delivery, as the acidic tumor microenvironment triggers enhanced release through PAMAM dendrimer chain relaxation, while minimizing release at physiological pH. Release patterns were similar for PAMAM-AuNC-DOX and its PEG-, FA-, and dual-modified systems, with first-order kinetics being most prominent at pH 4.5 (R^2^ up to 0.988). This implies that, independent of surface changes, the acidic environment enhanced protonation of the PAMAM dendrimer, leading to swelling and more consistent concentration-dependent release. The slightly higher *n* values observed at acidic pH quantitatively confirm this swelling contribution. The TPP^+^ conjugated nanocomplexes exhibited slightly altered release profiles, with TPP-PAM-AuNC-DOX showing the highest correlation with the Korsmeyer–Peppas model at pH 6.5 (R^2^ = 0.978), possibly due to the influence of TPP^+^ on matrix erosion. Interestingly, the PEG-modified nanocomplexes (standard and TPP^+^ conjugated) displayed improved zero-order kinetics (R^2^ up to 0.945) compared to their non-PEGylated counterparts, suggesting that PEGylation contributed to more sustained release patterns. This is consistent with the slightly lower *n* values observed for PEGylated formulations (0.37–0.49), indicating that PEG creates a more stable diffusion barrier. The FA-functionalized systems maintained strong first-order characteristics (R^2^ up to 0.984), indicating that FA conjugation did not significantly alter the fundamental release mechanisms. TPP-PEG-FA-PAM-AuNC-DOX exhibited the most consistent release profile (R^2^ = 0.968) at physiological pH, whereas all nanocomplexes showed diminished but significant correlations with first-order (R^2^ = 0.958–0.969) and Korsmeyer–Peppas (R^2^ = 0.961–0.971) models. In all FAuNC-DOX, the Higuchi model demonstrated a strong correlation (R_2_ = 0.941–0.978), indicating that diffusion was a secondary factor in drug release. Table 3 and Table 4 present the correlation coefficients for the tested models of the release profile of DOX-encapsulated nanoclusters.

### 2.5. Cellular Uptake and Subcellular Localization of Nanocomplexes

**Figure 8:**
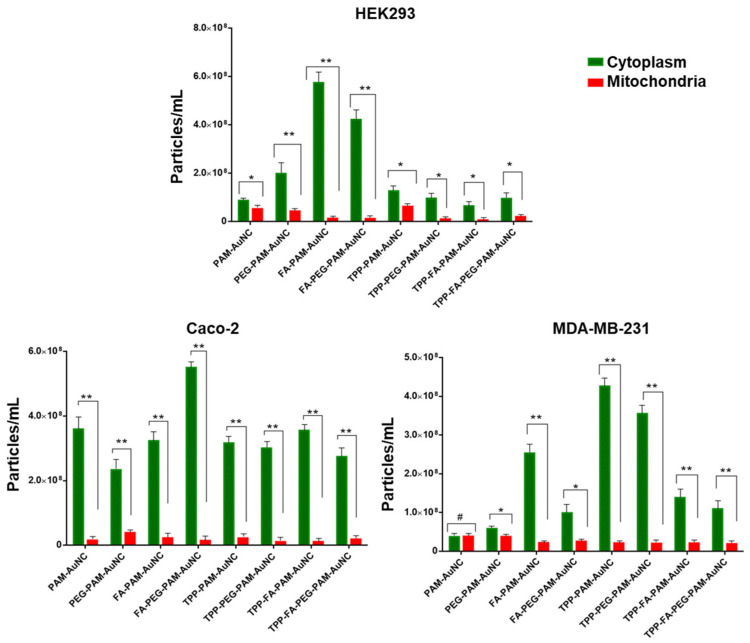
Cytosolic and mitochondrial distribution of functionalized AuNCs, as determined via NTA, for the HEK293, Caco-2, and MDA-MB-231 cells. Data are represented as the mean ± SD (*n* = 3). # = no statistical significance, statistical significance denoted as * = *p* < 0.05, ** = *p* < 0.01.

**Figure 9:**
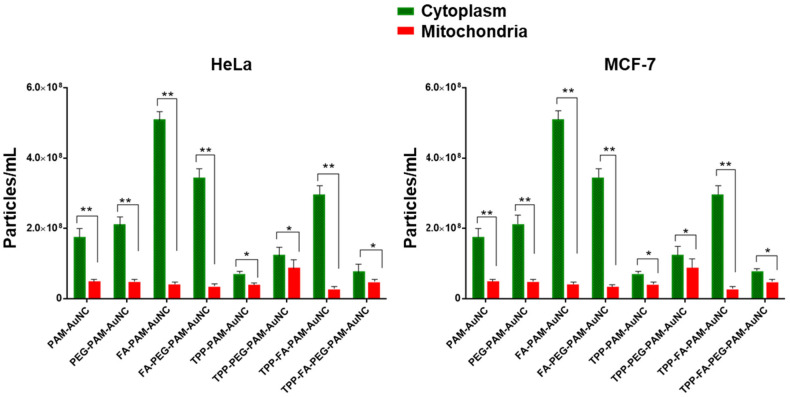
Cytosolic and mitochondrial distribution of functionalized AuNCs, as determined via NTA, for the HeLa and MCF-7 cells. Data are represented as the mean ± SD (*n* = 3). Statistical significance denoted as * = *p* < 0.05, ** = *p* < 0.01.

The distribution of the FAuNCs to the cytoplasm and mitochondria was measured using NTA in the Caco-2, HEK293, HeLa, MCF-7, and MDA-MB-231 cells. Figure 8 and Figure 9 illustrate the extent of mitochondrial and cytoplasmic uptake of the nanocomplexes. FA-conjugated AuNCs had better cytoplasmic accumulation (FA-PAM-AuNC: 3.2-fold and 2.5-fold) than the PAM-AuNCs in the MCF-7 and HeLa cells, which overexpress folate receptors. However, they showed low mitochondrial localization (<12%), suggesting FA-mediated endocytosis without mitochondrial affinity. However, TPP^+^-modified AuNCs demonstrated favorable mitochondrial localization, with TPP-PAM-AuNC achieving 62% mitochondrial localization in MCF-7 cells and 54% in HeLa cells. While FA boosted overall cellular uptake by 40% in the MCF-7 cells, mitochondrial localization decreased to 48%, indicating competition across uptake pathways. The TPP-FA-PAM-AuNC exhibited intermediate behavior. HeLa cells showed a high level of mitochondrial localization (36%). TPP-PEG-PAM-AuNCs demonstrated 15–20% lower mitochondrial accumulation than its non-PEGylated counterparts. Notably, cell-specificity was evident, with MDA-MB-231 cells showing the highest mitochondrial uptake of TPP-FA-PAM-AuNCs (55%) and Caco-2 the lowest (22%). Size-dependent effects were noted with AuNCs (~200 nm), where TPP-FA-PAM-AuNCs showed 30% lower total cellular uptake than the smaller TPP-PAM-AuNCs (~120 nm) in both MCF-7 and HeLa cells.

### 2.6. Cytotoxicity

**Figure 10:**
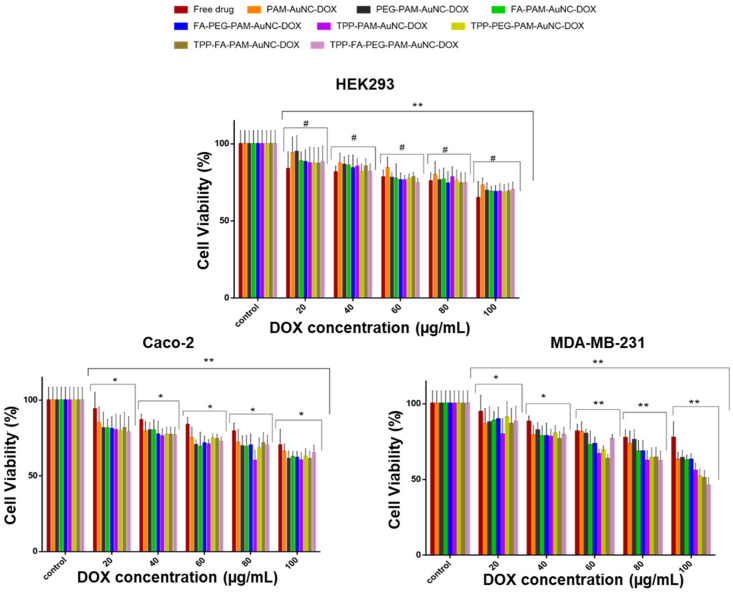
MTT cell viability assay of functionalized AuNC-DOX in the HEK293, Caco-2, and MDA-MB-231 cells. Data are represented as the means ± SD (*n* = 3) (statistical significance denoted as ** = *p* < 0.01, * = *p* < 0.05, # = no statistical significance). Control = untreated cells.

**Figure 11:**
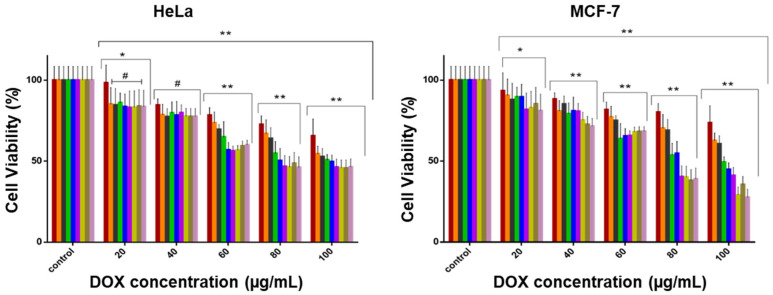
MTT cell viability assay in the HeLa and MCF-7 cells. Data are represented as the means ± SD (*n* = 3) (statistical significance denoted as * = *p* < 0.05, ** = *p* < 0.01, # = no statistical significance). Control = untreated cells.

The FAuNCs showed dose-dependent cytotoxicity across all cell lines (Figure 10 and Figure 11). Cell viability was greater than 90% at all dosages (20–100 µg/mL). The negligible toxicity of PAM-AuNCs (MCF-7: 91.7%; MDA-MB-231: 91.3%; Caco-2: 90.1% at 100 µg/mL) confirmed that its unaltered polymeric shell is biocompatible. At 100 µg/mL, PEGylation caused mild cytotoxicity (PEG-PAM-AuNC: 87.1% in Caco-2 against 93.1% in MCF-7), which may be a result of changed endocytic trafficking. However, FA-PEG-PAM-AuNCs demonstrated selective enhancement in the cancer cells (MCF-7: 93.8% vs. PEG-PAM-AuNC: 93.1% at 100 µg/mL), suggesting receptor-mediated specificity.

Folate conjugation (FA-PAM-AuNC, FA-PEG-PAM-AuNC) demonstrated comparable viability to their non-targeted counterparts. For TPP-PAM-AuNCs (100 µg/mL), viabilities of 89.7% in the MCF-7 and 91.2% in the MDA-MB-231 cells were noted, whereas TPP-PEG-PAM-AuNCs had slightly better cell viability in the Caco-2 cells (94.3%) compared to PEG-PAM-AuNCs (87.1%). Targeting ligand compensation mechanisms is suggested by the high viability maintained by the dual-ligand TPP-FA-PAM-AuNCs (HeLa: 90.1%; HEK293: 89.8%). The TPP-FA-PEG-PAM-AuNCs maintained biocompatibility (MCF-7: 92.0%; MDA-MB-231: 90.1%), suggesting PEG’s stabilizing effect. HEK293 demonstrated robustness (cell viability above 89.8% for all FAuNCs), but Caco-2 showed increased vulnerability to the PEGylated AuNCs.

The results for the DOX nanocomplexes are shown in Figure 10 and Figure 11. This study compared non-targeted and targeted (TPP^+^ and FA) nanocomplexes with free DOX, revealing distinct cytotoxicity patterns that emphasize the importance of ligand selection and cellular properties in shaping therapeutic outcomes [36]. All the nanocomplexes showed much better biocompatibility in the HEK293 cells than free DOX. At the highest concentration (100 μg/mL), viability ranged from 68.5% for TPP-FA-PEG-PAM-AuNC-DOX to 73.1% for PAM-AuNC-DOX, whereas free DOX showed much higher toxicity (65% viability). The protective effect was consistent across all concentrations, with some variation between 80 and 100 μg/mL; free DOX caused a 30–35% reduction in viability, compared to 15–25% for most nanocomplexes, in line with the literature [37,38].

MCF-7 cells demonstrated sensitivity to the dual-targeted TPP-FA-PEG-PAM-AuNC-DOX, achieving 27.5% cell viability at 100 μg/mL, outperforming all mono-targeted formulations (TPP-PAM-AuNC-DOX: 41.1%; FA-PAM-AuNC-DOX: 49.3%). This was a 2.7-fold increase in cytotoxicity compared with free DOX (73.6% viability). A threshold impact on optimal therapeutic action was suggested by the concentration-dependent response, with a noticeable decline in TPP-FA-PEG-PAM-AuNC-DOX viability between 60 and 100 μg/mL.

In the HeLa cells, the dual-targeted TPP-FA-PAM-AuNC-DOX achieved 45.7% viability at 100 μg/mL, compared to 50.7% for FA-PAM-AuNC-DOX and 65.6% for free DOX. The TPP^+^-conjugated compounds have higher cytotoxicity than their non-targeted counterparts, highlighting the benefit of mitochondrial targeting. Mitochondrial targeting was especially effective in the MDA-MB-231 cells. At the highest concentration, TPP-FA-PEG-PAM-AuNC-DOX (46.0%) and TPP-PAM-AuNC-DOX (55.9%) exhibited 1.4–1.7 times the cytotoxicity of the non-targeted PAM-AuNC-DOX (63.2%). A dose-response was seen for the MDA-MB-231 cells in contrast to the MCF-7 cells, where cytotoxicity rose linearly. In Caco-2 cells, cell viability for TPP^+^-conjugated nanocomplexes ranged from 61.3 to 66.3% at 100 μg/mL. Notably, PEGylation affected the cells differently. In HEK293 cells, decreased cytotoxicity at 100 μg/mL was observed compared to MCF-7 cells.

The IC_50_ (Table 5) values indicated that HEK293 cells had the highest tolerance (168.3–236.8 µg/mL), and HeLa cells showed the greatest sensitivity (45.1–74.8 µg/mL). The dual-targeted TPP-FA-PEG-PAM-AuNC-DOX exhibited exceptional potency in MCF-7 cells (45.4 µg/mL), representing a 2.6-fold improvement over the non-targeted PAM-AuNC-DOX (87.3 µg/mL) and 3.1-fold better than free DOX (126.4 µg/mL). The MDA-MB-231 cells showed intermediate sensitivity, with TPP-FA-PEG-PAM-AuNC-DOX (50.1 µg/mL) performing better than free DOX (206.4 µg/mL). Caco-2 cells also showed moderate sensitivity, with IC_50_ values ranging from 85.3 to 124.2 µg/mL, consistent with their lower receptor expression compared to MCF-7 cells. The superiority of the TPP^+^/FA dual-targeted nanocomplexes in all cells validated this targeting approach, with the most potent formulation (TPP-FA-PEG-PAM-AuNC-DOX) showing IC_50_ values 2.5–3.3 times lower than those of free DOX. These results explain the enhanced efficacy observed with mitochondrial/folate dual-targeting strategies.

**Figure 12:**
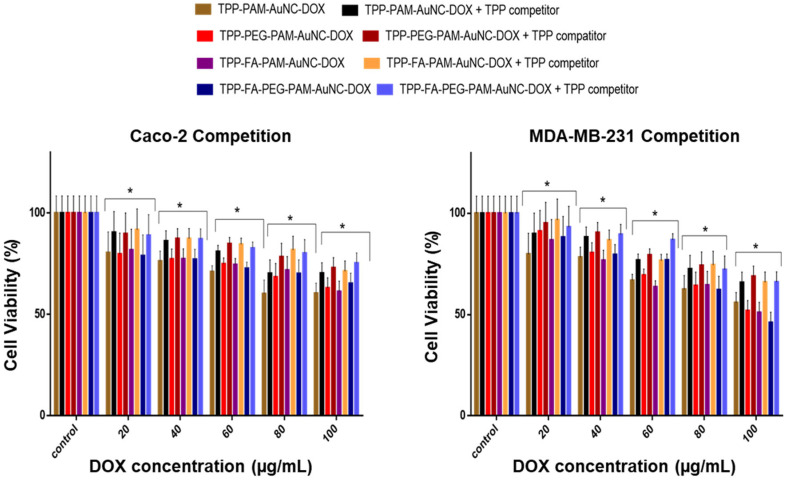
Competition assay of TPP^+^ functionalized AuNC-DOX on Caco-2 and MDA-MB-231 cells. TPP^+^ was added at an excess of 50× concentration. Data are represented as the mean ± SD (*n* = 3). Statistical significance is denoted as * = *p* < 0.05. Control = untreated cells.

**Figure 13:**
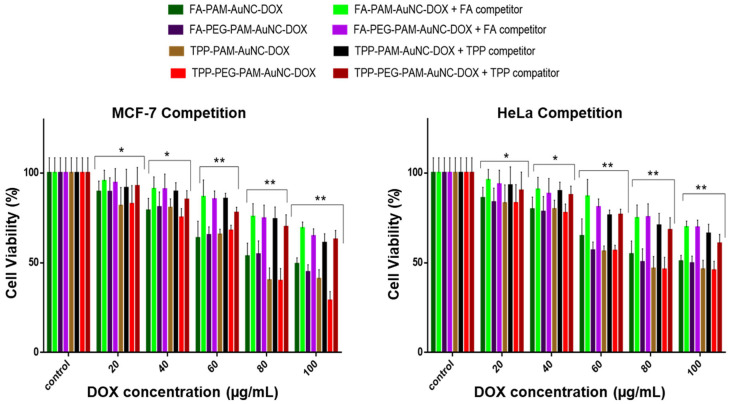
MTT competition assay cell viability studies of TPP^+^ and FA functionalized AuNC-DOX on MCF-7 and HeLa cells. TPP^+^ and FA were added at 50 times excess. Data are represented as the mean ± SD (*n* = 3). Statistical significance is denoted as * = *p* < 0.05, ** = *p* < 0.01. Control = untreated cells.

**Figure 14:**
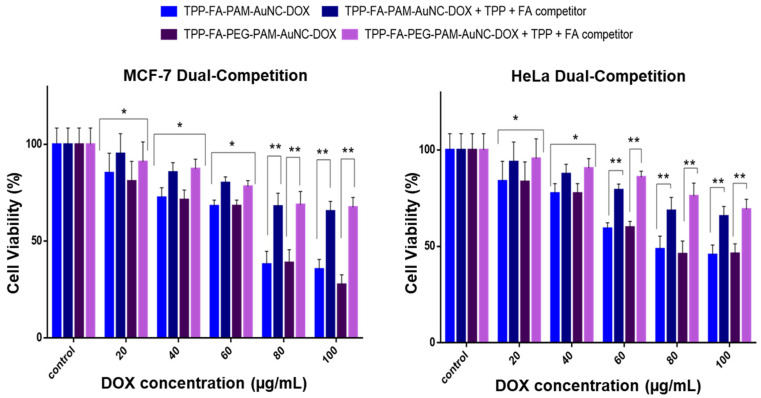
Dual competition assay cell viability studies of TPP-FA functionalized AuNC-DOX on MCF-7 and HeLa cells. FA was added at a concentration in excess of 50 times. Data are represented as the mean ± SD (*n* = 3). Statistical significance is denoted as * = *p* < 0.05, ** = *p* < 0.01. Control = untreated cells.

To corroborate receptor-mediated uptake and mitochondrial localization, a competition assay was performed (Figure 12, Figure 13 and Figure 14). Since cancer cells have a hyperactive mitochondrial metabolic rate, the TPP^+^ competition was used to assess any improved mitochondrial localization. The MCF-7 and HeLa cells, which are known to overexpress the folate receptor, were used to confirm receptor-mediated endocytosis. The excess FA added to the assay flooded and blocked the folate receptors on the cells, so the FA-targeted nanocomplexes could not access this pathway to enter the cells. Excess TPP^+^ would surround the mitochondrial membrane, reducing the uptake of TPP^+^-containing nanocomplexes. The results showed a cell viability of about 85% at 20 μg/mL, similar to the untargeted nanocomplexes, with a significant improvement in cell viability (* *p* < 0.05) at 80–100 μg/mL. At 80–100 μg/mL, cell viability significantly improved, and both cell lines exhibited viability above 65% (** *p* < 0.01), thus confirming that the receptor-mediated uptake of the FA-targeted nanocomplexes was further enhanced by the effect of TPP^+^ in inducing mitochondrial uptake.

The IC_50_ values for all FAuNC-DOX obtained in Table 6 for HEK293 cells were higher than the free drug IC_50_ values and were therefore not assayed further in vitro.

### 2.7. Mitopotential Assay

**Figure 15:**
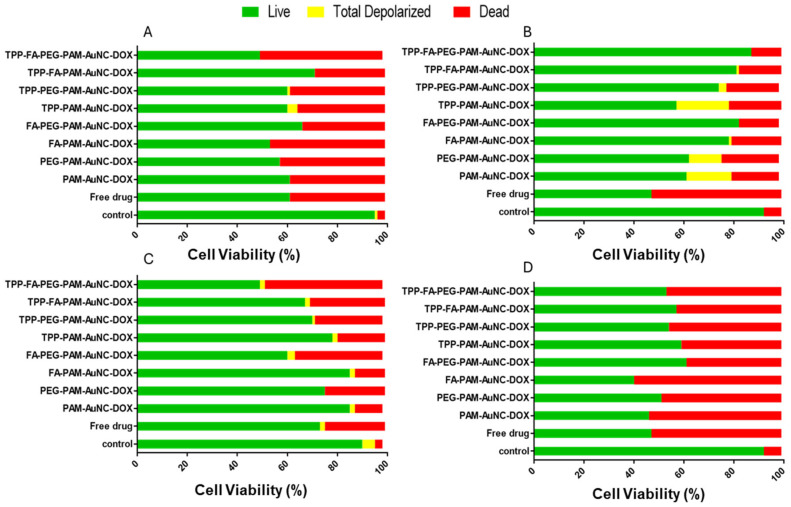
Graphical representations of the effect of targeted and untargeted nanocomplexes on the mitochondrial membrane potential in the (**A**) Caco-2, (**B**) MDA-MB-231, (**C**) HeLa, and (**D**) MCF-7 cells. Control = untreated cells.

Figure 15 presents a graphical representation of the mitochondrial effects in vitro, and Supplementary Figure S2 provides flow cytometry images. TPP-PAM-AuNCs induced 18% mitochondrial depolarization in the MDA-MB-231 cells, compared to the non-targeted PAM-AuNCs (1%). The TPP-PAM-AuNC-DOX caused greater depolarization (21%). Dual-targeted TPP-FA-PAM-AuNCs demonstrated better mitochondrial integrity in the MDA-MB-231 (1% depolarization vs. 21% for TPP-PAM-AuNC), indicating that the effects of TPP^+^ on the mitochondria may be modulated further by folate receptor-mediated uptake.

The Caco-2 cells showed varied responses, with only TPP-PAM-AuNCs inducing some depolarization (4%), while the other formulations had no effect. The significance of tumor biology in assessing the effectiveness of nanotherapeutics is evident in these cells, which had 92% cell viability in the controls compared to 49–71% after treatment. Although the Caco-2 cells exhibited little depolarization, the cytotoxicity observed with TPP-FA-PEG-PAM-AuNCs suggests that these cells may use alternative pathways to induce cell death. The TPP-FA-PEG-PAM-AuNCs induced 1–2% depolarization and up to 47% cell death in HeLa cells, indicating other modes of action, as HeLa cells displayed intermediate sensitivity.

Despite its high cytotoxicity (59% with FA-PAM-AuNC), MCF-7 cells showed no discernible depolarization with any treatment. Overall, three trends were seen in the data: (i) in the MDA-MB-231 cells, mitochondrial targeting correlated with efficacy (R^2^ = 0.89 between cell death and depolarization); (ii) Caco-2 and HeLa cells exhibited a minor association; and (iii) The MCF-7 cells showed that these pathways were independent.

The greater depolarization with TPP-PAM-AuNCs in MDA-MB-231 cells than in MCF-7 cells at equivalent doses suggests that mitochondrial targeting may depend on the cancer cell type. The low impact of the FA-targeted nanocomplexes in MDA-MB-231 cells implied that receptor-mediated endocytosis could occasionally avoid mitochondrial involvement. PEGylated nanocomplexes were also cell-specific, with good cell viability in the MDA-MB-231 cells (87% for TPP-FA-PEG-PAM-AuNC and 57% for the TPP-PAM-AuNC).

### 2.8. Caspase 3/7 Activity

**Figure 16:**
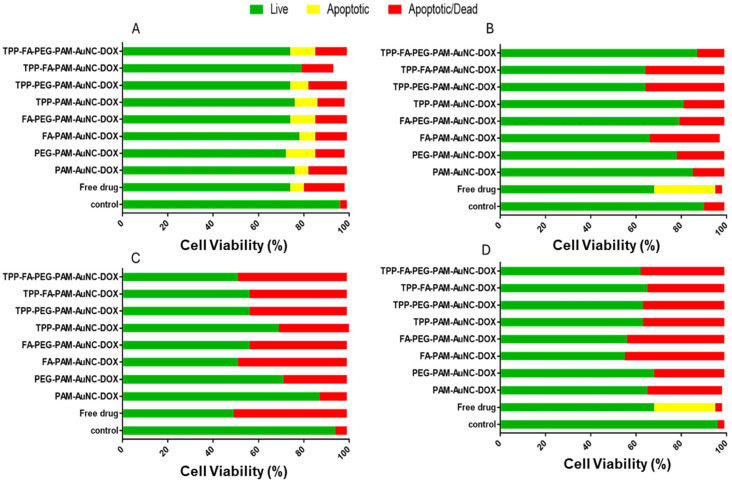
Graphical representations of the effect of the targeted and untargeted nanocomplexes on caspases 3/7 activity in (**A**) Caco-2, (**B**) MDA-MB-231, (**C**) HeLa, and (**D**) MCF-7 cells. Control = untreated cells.

Figure 16 presents a graphical representation of caspase activation in vitro, with Supplementary Figure S3 providing the corresponding flow cytometry images.

Free DOX exhibited the highest apoptotic induction in HeLa cells (50%), the non-targeted nanocomplexes showed low activity (PAM-AuNC = 12%), and targeted formulations showed moderate activity (TPP-FA-PAM-AuNC 43%; FA-PAM-AuNC 48%). The over 2-fold difference between free DOX and TPP-FA-PAM-AuNCs suggested partial circumvention of standard apoptotic pathways via nano-encapsulation, consistent with HeLa’s known susceptibility to mitochondria-mediated apoptosis. Free DOX induced early apoptosis (27% apoptotic cells) rather than late-stage cell death (3% apoptotic/dead). All nanocomplexes generated late-apoptotic/dead populations (33–44%) in MCF-7 cells. Caco-2 cells exhibited moderate apoptotic responses with the TPP-PAM-AuNCs showing early/late apoptosis (10% apoptotic, 12% apoptotic/dead) and PEG-PAM-AuNCs producing early apoptosis (13%). These dual-targeted nanocomplexes appeared to preferentially trigger non-apoptotic pathways in Caco-2 cells, as evidenced by the absence of apoptotic cells in TPP-FA-PAM-AuNC-treated cells (79% live, 14% apoptotic/dead). In MDA-MB-231 cells, TPP-FA-PEG-PAM-AuNCs showed protective effects (87% viable, 12% apoptotic/dead), whereas FA-PAM-AuNCs generated late apoptosis (31%), which was almost identical to the effects of free DOX (27% apoptotic, 3% apoptotic/dead). Three major mechanistic insights emerge from the results. Firstly, mitochondrial targeting (via TPP^+^) enhances late apoptosis in susceptible cells (HeLa: 43%), but not in caspase-3-deficient MCF-7 cells, confirming the essential role of this enzyme in the intrinsic apoptotic pathway. Secondly, targeting the folate receptor had the opposite effect in HeLa cells (FA-PAM-AuNC: 48%) and MDA-MB-231 cells (FA-PAM-AuNC: 31% vs. FA-PEG-PAM-AuNC: 20%), indicating that receptor expression levels significantly influenced the results.

PEGylation slowed intracellular drug release, thereby reducing apoptotic induction (PEG-PAM-AuNC: 28% vs. PAM-AuNC: 12%) in HeLa cells. Although the apoptotic induction of TPP-FA-PAM-AuNC-DOX (50%) was lower than that of free DOX (67%), it demonstrated greater cytotoxicity (52.81%) in the HeLa cells, suggesting that these nanocomplexes caused cell death via both caspase-dependent and -independent pathways. The over 8-fold caspase induction of PAM-AuNC-DOX over free DOX in the MCF-7 cells points to the activation of compensatory executioner caspases (possibly caspase-7).

### 2.9. Apoptosis Induction: Dual Fluorescence Assay

**Figure 17:**
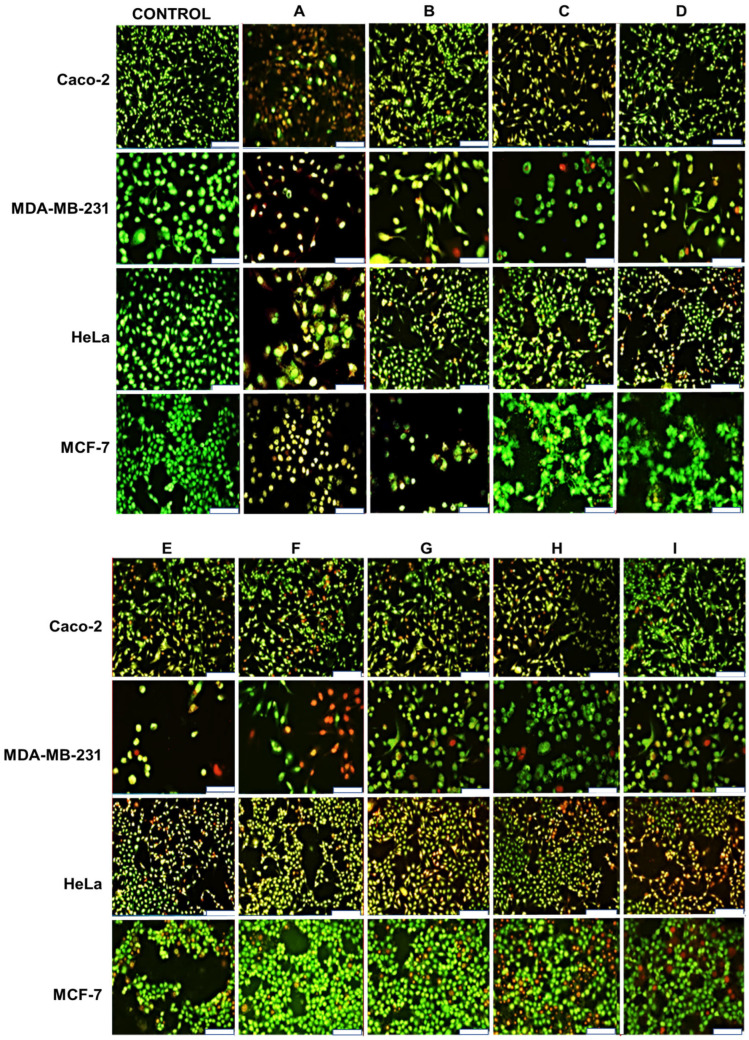
Fluorescent images of acridine orange/ethidium bromide-stained cells showing apoptosis induction by FAuNC-DOX in the Caco-2, MDA-MB-231, HeLa, and MCF-7 cells. Control = untreated cells, (**A**) free DOX treatment, (**B**) PAM-AuNC-DOX treatment, (**C**) PEG-PAM-AuNC-DOX treatment, (**D**) FA-PAM-AuNC-DOX treatment, (**E**) FA-PEG-PAM-AuNC-DOX treatment, (**F**) TPP-PAM-AuNC-DOX treatment, (**G**) TPP-PEG-PAM-AuNC-DOX treatment, (**H**) TPP-FA-PAM-AuNC-DOX treatment, and (**I**) TPP-FA-PEG-PAM-AuNC-DOX treatment. Scale bar = 100 μm.

To further evaluate the effect of the targeted nanocomplexes against free DOX, a qualitative fluorescence analysis was performed to visualize the degree of apoptosis in selected cell lines. Cells were stained with acridine orange/ethidium bromide (AO/EB) after being incubated for 24 h with DOX and the nanocomplexes at a predetermined concentration [33]. EB enters dead cells when the membrane potential is lost and interacts with fragmented DNA, producing orange fluorescence, while AO is cell-permeable and interacts with double-stranded DNA in viable cells, producing green fluorescence [39]. In the cancer cells, various cytomorphological alterations suggestive of apoptosis were evident (Figure 17). Morphological alterations, including cell shrinkage and membrane blebbing, were noted. These alterations were severe in cells exposed to nanocomplexes, but modest in cells treated with DOX only.

### 2.10. Oxidative Stress Assay

**Figure 18:**
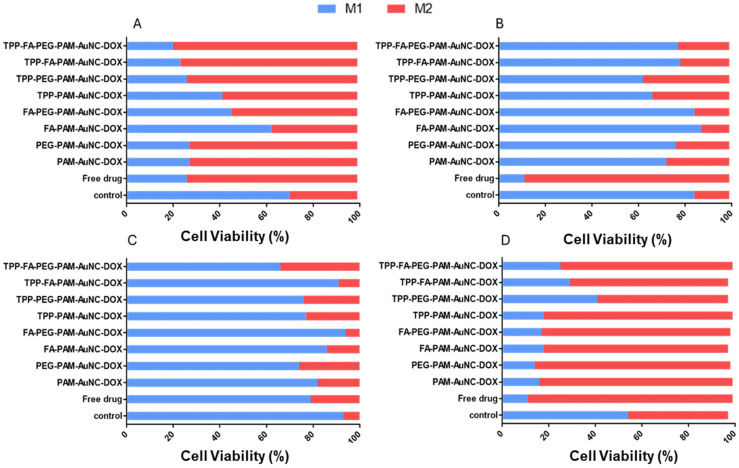
Graphical representations of the effect of targeted and untargeted nanocomplexes on oxidative stress in (**A**) Caco-2, (**B**) MDA-MB-231, (**C**) HeLa, and (**D**) MCF-7 cells. M1 = ROS negative, M2 = ROS positive. Control = untreated cells.

The treatments produced variations in the cells’ ROS-mediated responses (Figure 18 and Supplementary Figure S4). TPP-FA-PEG-PAM-AuNCs caused the most oxidative stress in the Caco-2 cells (79% M2 high-ROS population against 29% in controls), followed by TPP-FA-PAM-AuNCs (76% M2) and TPP-PAM-AuNCs (58% M2). In these cells, the FA-PAM-AuNCs showed a beneficial antioxidant effect (23% M2, compared with 72–73% for the non-targeted formulations), indicating that activation of the folate receptor may trigger compensatory redox regulatory processes. Compared to free DOX (73% M2), the number of ROS-positive cells after TPP-FA-PEG-PAM-AuNC treatment increased 3.4-fold.

The ROS production in the MCF-7 cells was more pronounced. The greatest percentage of M2 cells generated by free DOX was 88%, followed by TPP-FA-PEG-PAM-AuNCs (74%), TPP-FA-PAM-AuNCs (68%), and TPP-PEG-PAM-AuNCs (56%). This indicated a dose-dependent ROS generation by the targeted nanocomplexes. HeLa cells displayed a different profile, with the majority of treatments showing lower levels of oxidative stress than the controls (34% M2 baseline). While targeted formulations had moderate benefits (TPP-FA-PEG-PAM-AuNC: 7% M2; TPP-FA-PAM-AuNC: 11% M2), free DOX reduced high-ROS populations to 21% M2. In MDA-MB-231 cells, free DOX induced the highest oxidative stress (88% M2), while FA-PAM-AuNCs showed strong protection (12% M2) compared with the control (15%).

## 3. Discussion

The growing interest in the green synthesis of NPs, driven by their expanding use in clinical studies, emanates from their minimal environmental toxicity and straightforward production methods. The green synthesis of inorganic NPs utilizes the reducing capabilities of bio-extracts to convert metallic salts from their oxidized state to the zero-oxidation state [40]. These NPs inherit distinctive characteristics from the bio-extracts used in their formulations. We successfully produced AuNCs and modified them with PAMAM, folate, TPP^+^, and PEG. Dendrimer modification enhanced their stability and introduced important functional groups for further modifications.

The SPR of the AuNCs was consistent with the literature [16,32]. The addition of TPP^+^ altered the optical properties, with TPP^+^-functionalized AuNCs exhibiting distinct absorbance patterns. The PEG and FA affected the optical properties and structure of the AuNCs, leading to changes in absorbance [41]. FTIR data provided compelling evidence for molecules with aliphatic chains, aromatic or conjugated systems, and hydroxyl/amine groups. The observed spectrum properties are largely influenced by hydrogen bonding. Since each AuNC derivative has undergone unique modifications (PEGylation, FA conjugation, TPP^+^ functionalization), the variances in transmittance reflected the differences in their chemical compositions, confirming successful functionalization of the AuNCs [42].

While charged NPs can interact indiscriminately with biomolecules, cationic NPs show enhanced cellular membrane association and uptake compared to anionic NPs [43]. The recorded zeta potentials suggested relative stability of the nanocomplexes, implying that they might comprehensively influence interactions with cellular and mitochondrial membranes, given their negative membrane potential (Ψ). PAM-AuNCs were large, indicating a water-retaining PAMAM corona [44]. The nanocomplexes showed little change in particle size after DOX binding and no apparent signs of instability or aggregation. These findings also aligned with the literature [45]. Overall, the sizes from TEM and NTA differed considerably. This was due to the preparation techniques used for each assay. TEM measures the dry size of the NPs, whereas NTA examines them in an aqueous environment, as would be observed in an in vivo system [38]. Hence. The NTA hydrodynamic size was considered for the studies conducted. The zeta potential reflects the NP’s surface charge relative to the conducting fluid and serves as an indicator of NP stability and biomolecular interactions [46]. Good stability has been associated with ζ potentials of ±20 mV or greater [38].

The high DOX encapsulation efficiency for all nanocomplexes (>78%) was consistent with reports in the literature for other metal NPs, although slightly higher [46,47]. This could be attributed to the encapsulation being done during the synthesis. Drug release profiles demonstrate how these drug formulations behave over time at various pH levels [48]. Although surface modifications affected release rates, they did not significantly alter the underlying kinetic mechanisms, as observed in earlier reports [46,47,49]. These results demonstrate the potential of these nanocomplexes as drug-delivery vectors and are consistent with other drug-release profiles reported in the literature [50]. Overall, the first-order and Korsmeyer–Peppas models were the most accurate for predicting drug release from the nanocomplexes, regardless of the composition. These models indicated a concentration-dependent kinetics with a mix of diffusion and erosion mechanisms dominating drug release [51].

The preliminary cellular uptake and localization data suggest possible ligand-dependent targeting, cell-specificity, and size- and charge-dependent uptake [52]. The mitochondrial localization in HeLa cells has been reported to be due to differences in receptor recycling kinetics [53]. The results of steric hindrance by PEG were evident in the uniform decrease in mitochondrial uptake in all cells [54]. This stabilization was clearly evident in breast cancer cells, as shown in a recent study [55]. These findings confirm the safety of functionalized AuNCs as drug delivery vehicles with high cell viability.

The concentration-dependent cytotoxicity of the FAuNCs was consistent with the literature [56]. The high cytotoxicity of TPP-FA-PEG-PAM-AuNC-DOX in MCF-7 cells was likely due to the TPP^+^ accumulation and FA-mediated receptor internalization [57], as well as pH-triggered drug release in the acidic tumor microenvironment [58]. The epithelial barrier characteristics and distinct receptor expression patterns in Caco-2 cells, in contrast to those of other cancer cell lines, may account for their high sensitivity [59]. PEGylation affected the cells differently. PEG’s steric effects may prolong circulation time while impeding uptake in healthy cells [60], as seen in HEK293 cells.

The cell-specific responses reflect the differences in folate receptor density and mitochondrial membrane potential, with the low IC_50_ in the MCF-7 cells suggesting both high folate receptor expression and a possible mitochondrial vulnerability. The favorable activity of dual-targeted formulations supports previous findings that TPP^+^-functionalization can effectively localize in mitochondria via electrostatic interactions with the negatively charged membrane, while folate targeting provides additional uptake via receptor-mediated endocytosis. Recent research on AuNC systems has shown that this combination approach can effectively accumulate drugs in the mitochondrial and cytoplasmic compartments of cancer cells. because of the negatively charged mitochondrial membrane’s electrostatic pull [61].

The anticancer mechanisms of DOX include mitochondrial dysfunction, the production of reactive oxygen species (ROS), DNA intercalation, and topoisomerase II inhibition [62,63,64]. Depolarization of the mitochondrial membrane greatly increases its lethal potential. Since cytotoxicity and mitochondrial effects are unrelated, it is quite likely that DOX’s main DNA-targeting mechanisms predominate over mitochondrial routes in some types of cancers. However, different pathways may serve as potential mechanisms of cell death and thus need to be investigated further. The mitopotential assay results are consistent with the concept that DOX’s effects are cancer-specific and possibly mediated by both mitochondrial-dependent and -independent mechanisms [65]. The high depolarization for TPP^+^-conjugates is consistent with cancer mitochondria’s known hyperpolarized condition [66], making them vulnerable to TPP^+^-mediated targeting. The lack of action in MCF-7 cells supports the involvement of other mechanisms, even though TPP^+^ conjugation did show improvements in mitochondrial targeting [67,68]. According to [69], this dual-pathway approach explains clinical data showing varying DOX sensitivity and raises the possibility that assessing the mitochondrial profile pre-treatment could predict therapy effectiveness. Cell-specific differences can reflect existing clinical variations [70]. The significant difference in TPP-FA-PAM-AuNC efficacy between Caco-2 cells and MDA-MB-231 cells highlights the need for customized approaches.

The apoptotic cascade following mitochondrial membrane disruption involves the release of cytochrome c and the subsequent activation of executioner caspases 3/7 [71]. Cell line preferences were also noted for the caspase activity, highlighting the underlying dependence of nanocomplex effects on signaling pathways, particularly in different cancer types [72]. As expected, given their caspase-3 deficiency [73], MCF-7 cells generated late-apoptotic/dead populations, suggesting the presence of alternative mechanisms of cell death that bypass the traditional apoptotic pathway [74]. Cell line variations mirror clinical heterogeneity in the chemotherapy response, underscoring the need for personalized approaches based on tumor protease profiles. The dual-staining method differentiates between cells at early or late stages of apoptosis, with light-yellow fluorescence indicating early apoptosis and orange fluorescence indicating late apoptosis [39]. According to the earlier tests, the TPP-FA-PAM-AuNC-DOX nanocomplexes had the greatest overall effect on the cells. The significance of these nanocomplexes in improving drug bioavailability and pharmacokinetics was demonstrated by the overall efficiency of the FA-TPP^+^-nanocomplexes compared with free DOX.

ROS are important mediators of p53 activation and cell cycle arrest, demonstrating the well-established link between oxidative stress and cell cycle control [75,76]. The number of high-ROS positive cells after treatment with TPP-FA-PEG-PAM-AuNCs highlighted the benefit of mitochondrial targeting for ROS formation [77]. The TPP-PEG-PAM-AuNC’s activity in MCF-7 cells (41% M1, compared with 18–29% for other TPP^+^ containing nanocomplexes) may be related to PEG-mediated scavenging of mitochondrial ROS, underscoring the intricate relationship between oxidative effects and surface modification [78]. The lower oxidative stress in HeLa cells suggests that they may have stronger antioxidant defenses that are triggered differently by various therapies. The intermediate effects of the TPP^+^ formulations in HeLa cells reduced ROS production to a limited extent. These trends are also consistent with breast tumors’ recognized metabolic variability and fluctuating vulnerability to oxidative stressors [79]. There are three possible mechanistic revelations. Mitochondrial and FR targeting may have modulated ROS production, decreasing oxidative stress in Caco-2 cells and increasing it in MCF-7 cells. PEGylation coupled with TPP^+^ produced increased ROS production in Caco-2 cells but decreased it in MCF-7 cells. Lastly, the antioxidant capacities of different cells led to distinct therapeutic outcomes, with HeLa cells being the most resistant to ROS-mediated effects and MCF-7 cells the most susceptible.

## 4. Materials and Methods

### 4.1. Materials

Gold (III) chloride trihydrate (HAuCl_4_.3H_2_O), glutathione, phosphate-buffered saline tablets [PBS, (140 mM NaCl, 10 mM phosphate buffer, 3 mM KCl)], 3-[(4,5-dimethylthiazol-2-yl)-2,5-diphenyl tetrazolium bromide] (MTT), ethidium bromide, glacial acetic acid, dimethyl sulfoxide [DMSO], and doxorubicin hydrochloride (DOX) (Mw: 579.98 g.mol^−1^) were supplied by Merck (Darmstadt, Germany). Polyamidoamine (PAMAM dendrimer, ethylenediamine core, generation 5.0) dendrimers (Mw: 28,824.81 g/mol) (PAM)], sodium tripolyphosphate (STPP), acridine orange hemi (zinc chloride) salt [3,6-bis(dimethylamino) acridine hydrochloride zinc chloride double salt], maleimide-polyethylene glycol-N-hydroxysuccinimide (PEG), 1-ethyl-3-(3-dimethylamino propyl) carbodiimide (EDC) N-hydroxysuccinimide, (NHS), folic acid (FA), triphenylphosphonium (TPP^+^), and dialysis tubing (MWCO = 2000 Daltons (Da)) were supplied by Sigma-Aldrich Chemical Co. (St. Louis, MO, USA). Muse^®^ mitopotential, Caspase-3/7 assay, and Oxidative stress Assay kits were purchased from Luminex Corporation (Austin, TX, USA). The human embryonic kidney (HEK293), colon adenocarcinoma (Caco-2), cervical carcinoma (HeLa), epithelial metastatic mammary adenocarcinoma (MDA-MB-231), and breast adenocarcinoma (MCF-7) cells were originally obtained from the American Type Culture Collection (ATCC), Manassas, VA, USA. Cells were routinely tested for mycoplasma before use. Sterile fetal bovine serum (FBS) was supplied by Hyclone GE Healthcare (South Logan, UT, USA). Eagle’s Minimum Essential Medium (EMEM) with L-glutamine, penicillin/streptomycin/amphotericin B (100×) antibiotic mixture, and trypsin-versene-EDTA mixture were supplied by Lonza Bio-Whittaker (Verviers, Belgium). All sterile tissue culture plasticware was sourced from Corning Inc. (New York, NY, USA). Reagents were all of analytical grade, and ultrapure (18 MOhm) water (Milli-Q Academic, Millipore, Molsheim, France) was used throughout.

### 4.2. Synthesis of Gold Nanoclusters (AuNCs) and PAMAM-Functionalized AuNCs (PAM-AuNCs)

AuNCs were synthesized through the reduction of gold (III) chloride trihydrate (HAuCl_4_3H_2_O), with glutathione (GSH) [16,32]. Briefly, 0.50 mL of a freshly prepared aqueous solution of HAuCl_4_.3H_2_O (20 mM) and 0.15 mL of GSH (100 mM) were mixed in 4.35 mL ultrapure H_2_O at 25 °C. The reaction mixture was heated in a glycerol bath to 70 °C with gentle stirring (500 rpm) for 24 h to avoid evaporation. A color change from clear to a deep yellow indicated the successful formation of the AuNCs. The solution was in the dark at 4 °C due to the light sensitivity of the AuNCs [80]. The final theoretical concentration of the AuNC solution was 1.58 mM (0.17 mg/mL). This was utilized for the functionalization with PAMAM and the conjugation of DOX.

### 4.3. Encapsulation of DOX and Formation of Functionalized AuNC-DOX Nanocomplexes

Approximately 5 mL of the AuNCs was added to 0.5 mL of sodium tripolyphosphate (STPP, 2 mM in 1% acetic acid), and the mixture was stirred for 4 h. DOX was incorporated by adding 0.5 mL of DOX (2 mg/mL) to 1.5 mL of the AuNC-STPP solution, stirring for 2 h. This produced the AuNC-DOX nanoconjugate.

To formulate the PAMAM-containing nanocomplex (PAM-AuNC-DOX), 1 mL of PAMAM (1 mg/mL) was activated for 2 h with 1 mL of NHS/EDC, followed by the addition of 1 mL of AuNC-DOX dropwise with stirring for 24 h. The solution was then dialyzed (MWCO = 2000 Da) against 18 MΩ water for 12 h. PEGylation was achieved by adding 90 μL of PEG (1 mg/mL) to 1 mL of PAMAM (1 mg/mL) solution, which was mixed for 2 h. Approximately 1 mL of the AuNC-DOX solution was then added dropwise, and the mixture was stirred for 24 h.

FA-PAM-AuNC-DOX was formulated by mixing 1 mL of FA (0.5 mg/mL in DMSO) with 1 mL of PAMAM (1 mg/mL) and activating it for 2 h with NHS/EDC. Thereafter, 1 mL of AuNC-DOX was added dropwise. The solution was stirred for 24 h, then dialyzed as previously described. To formulate FA-PEG-PAM-AuNC-DOX, 1 mL of FA (0.5 mg/mL in DMSO) and 90 μL of PEG (1 mg/mL) were mixed with 1 mL of PAMAM and activated for 2 h with NHS/EDC, followed by the dropwise addition of AuNC-DOX (1 mL). The solution was stirred for 24 h and dialyzed as described earlier.

To formulate the triphenylphosphonium cation (TPP^+^) containing PAM-AuNC-DOX, 1 mL of TPP^+^ (0.7 mg/mL) was mixed with 1 mL of PAMAM (1 mg/mL) and activated for 2 h with NHS/EDC. AuNC-DOX (1 mL) was then added dropwise, mixed for 24 h, and dialyzed as previously described. The TPP-PEG-PAM-AuNC-DOX was formulated by mixing 1 mL of TPP^+^ (0.7 mg/mL) and 90 μL of PEG (1 mg/mL) with 1 mL of PAMAM and then activating it with NHS/EDC as previously described. Thereafter, 1 mL of AuNC-DOX was added, and the solution was stirred for 24 h and dialyzed as previously.

TPP-FA-PAM-AuNC-DOX was formulated by mixing 1 mL of TPP^+^, 1 mL of FA (0.5 mg/mL in DMSO), and 1 mL of PAMAM. The mixture was then activated as described earlier, followed by the dropwise addition of 1 mL of AuNC-DOX. The solution was stirred for 24 h and dialyzed as previously described. TPP-FA-PEG-PAM-AuNC-DOX was formulated by mixing 1 mL of TPP^+^, 1 mL of FA (0.5 mg/mL in DMSO), 90 μL of PEG (1 mg/mL), and 1 mL of PAMAM. The mixture was then activated by adding AuNC-DOX (1 mL) dropwise. The solution was stirred and dialyzed as previously described.

### 4.4. Characterization of AuNCs and Their Nanocomplexes

#### 4.4.1. Ultraviolet-Visible (UV-Vis) and Fourier Transform Infra-Red (FTIR) Spectroscopy

UV-vis spectroscopy (Jasco V-730 Bio-spectrophotometer, Tokyo, Japan) was used to determine the absorbance spectra of all AuNCs and their nanocomplexes with DOX. The successful synthesis of the AuNCs was confirmed by absorbance in the 250–400 nm range, with shifts in the peak indicating successful functionalization with PAMAM, PEG, FA, TPP^+^, or conjugation with DOX.

FTIR served to verify the presence of essential bonds and functional groups in the AuNCs. The samples were analyzed using a PerkinElmer Spectrum spectrophotometer (Waltham, MA, USA), and IR spectra from 4000 cm^−1^ to 380 cm^−1^ were obtained using Spectrum Analysis Software 10.

#### 4.4.2. Nanoparticle Tracking Analysis (NTA)

NTA determines the hydrodynamic size, concentration, and zeta potentials of nanoparticles [81] and was conducted using a Nanosight NS-500 (Malvern Instruments, Worcestershire, UK) operating at 25 °C. Samples (1 mL) were analyzed in triplicate to ensure the validity of the results obtained.

#### 4.4.3. Transmission Electron Microscopy (TEM)

TEM was used to assess the ultrastructural morphology of all AuNCs and their nanocomplexes. Approximately 10 µL of each sample was added to a 400-mesh carbon-coated copper grid (Ted Pella Inc., Redding, CA, USA), air-dried at room temperature, and viewed using a JEOL-JEM T1010 electron microscope (Jeol, Tokyo, Japan) at 60,000× magnification and a 100 kV acceleration voltage. The TEM images were captured and analyzed using the instrument-associated iTEM Soft Imaging Systems (SIS) Megaview III fitted with a side-mounted 3-megapixel digital camera.

### 4.5. Drug Encapsulation and Release Studies

#### 4.5.1. Drug Encapsulation Efficiency

The AuNC-DOX nanocomplex was vortexed and sonicated for 15 min before absorbance was measured at 300 nm to determine the total DOX present in the sample [82]. The sample was then centrifuged at 2000 rpm for 15 min to separate the AuNC-DOX nanocomplex from the free unbound drug. The supernatant was removed, and the absorbance of the unbound drug was measured using a Jasco V-730 Bio-spectrophotometer (Tokyo, Japan). The encapsulation efficiency (EE) and drug loading (DL) of the nanocomplex AuNC-DOX were determined using Equations (1) and (2) [83].

(1) $$EE\left(\%\right)=\frac{Total\;DOX-Unbound\;DOX}{Total\;DOX}\times 100$$

(2) $$DL\left(\mu g/mg\right)=\frac{Encapsulated\;DOX\;(\mu g)}{Mass\;of\;nanocarrier\;(mg)}$$

#### 4.5.2. Drug Release

The amount of drug released from all nanocomplexes was determined over 48 h at pH 4.5, 6.5, and 7.4 to assess the pH-responsive behavior of the nanocomplexes [84]. Approximately 1 mL of each nanocomplex was dialyzed (1000-Da MWCO) against PBS (5 mL) at the respective pHs. Absorbance readings (300 nm) of the PBS (250 µL) were taken at 4 h intervals over 48 h. An equivalent amount of PBS was replaced to maintain the sink volume. A drug release profile was generated at each pH using the amount of drug encapsulated within the nanocomplex as a reference.

#### 4.5.3. Drug Release Kinetics

Table 6 lists the models that were used to determine the release kinetics of DOX from the nanocomplexes. Drug concentrations M_0_ and M_t_ indicate the starting concentration and cumulative quantity released at time t, respectively, while drug fraction M_t_/M_∞_ indicates the amount released at time t. The rate constant k corresponds to the models mentioned, and *n* represents the diffusional exponent, which indicates the drug release mechanism.

**Table 6:** Kinetic models used to assess the drug release mechanism from the nanocomplexes.

Kinetic Model	Equation	Reference
Zero-order	M_t_ = M_0_ + k_0t_	[85]
First order	logM_t_ = log M_0_ + k_t_/2.303	[86]
Higuchi	Mt = k_H_√t	[87]
Hixson–Crowell	(M_t_ − M_∞_)^1/3^ = k_HC_⋅t	[88]
Korsmeyer–Peppas	M_t_/M_∞_ = k_kp_.t^n^	[89]

### 4.6. In Vitro Mitochondrial Localization Studies

The HEK293, Caco-2, MDA-MB-231, HeLa, and MCF-7 cells were plated in 75 cm^2^ flasks at a density of 2.0 × 10^5^ in 25 mL EMEM and incubated overnight at 37 °C in 5% CO_2_. After replacing the medium, the cells were exposed to 1.58 mg/mL of each FAuNC at 37 °C for 12 h. The cells were then washed three times with cold PBS (pH 7.4), trypsinized, and centrifuged at 300× *g* for 5 min at 4 °C. The pellet was lysed in 10% cell lysis buffer (50 µL) on ice for 5 min, with intermittent vortexing. Subsequently, 450 µL of 0.25 M sucrose buffer (pH 7.4) was added, and the suspension was centrifuged twice at 700× *g* at 4 °C to remove cellular debris. The supernatant was then centrifuged at 12,000× *g* for 10 min at 4 °C to separate the mitochondrial fraction (pellet) from the cytoplasmic fraction (supernatant). The mitochondrial fraction was washed in sucrose buffer at 10,000× *g* for 10 min at 4 °C. Both fractions were then digested in aqua regia for 2 h at 90 °C and analyzed via NTA to determine the FAuNC uptake concentrations.

### 4.7. In Vitro Cytotoxicity

The 3-(4, 5-dimethylthiazolyl-2)-2, 5-diphenyltetrazolium bromide (MTT) assay was used to evaluate the cytotoxicity of the nanocomplexes in vitro. The cells were seeded into 96-well plates containing 100 μL of growth medium, at a seeding density of 2.5 × 10^4^ cells/well, and incubated for 24 h at 37 °C. The medium in the wells was then replenished, followed by the introduction of FAuNCs and FAuNC-DOX in triplicate at 20, 40, 60, and 100 µg. Free DOX was included at the theoretical amounts within the nanocomplex (free DOX concentration was normalized to the amount of DOX encapsulated within FAuNC) to ensure an accurate comparative study. A competition assay was conducted for drug nanocomplexes to assess FA receptor-mediated endocytosis (RME) and TPP^+^ induced mitochondrial localization. For the competition assay, cells were flooded with excess FA and TPP^+^ at a 50× concentration of each and incubated for 1 h before addition of the targeted nanocomplexes. The cells were incubated for 48 h at 37 °C, after which the medium was replaced with fresh growth medium (100 µL) containing 10 µL of MTT stock (5 mg/mL in PBS) and incubated for 4 h at 37 °C [90,91]. This medium/MTT mixture was then removed, and the resultant formazan crystals were solubilized in 100 µL of DMSO. The optical densities (ODs) were determined at 540 nm using a Mindray MR-96A microplate reader (Vacutec, Hamburg, Germany), with DMSO as the blank. Cell viabilities (%) were calculated using Equation (3).

(3) $$Cell\;Viability\left(\%\right)=\frac{OD\;treated\;cells}{OD\;control\;cells}\times 100$$

### 4.8. Mitopotential Assay

Cells were seeded in a 24-well plate at a density of 4.5 × 10^5^ cells/well and treated with the drug and nanocomplexes at their IC_50_ values. After a 48 h incubation, the cells were washed with PBS, scraped from the wells, and resuspended in 100 µL of 1 × BA assay buffer. Approximately 95 µL of Muse^®^ Mitopotential working solution (Mitopotential reagent 1:1000 1× BA assay buffer, Luminex, Austin, TX, USA) was added to each tube, and cells were incubated at 37 °C for 20 min. Thereafter, 5 µL of 7-aminoactinomycin D (7-AAD) was added, followed by incubation at room temperature for 5 min. Mitochondrial membrane potential was assessed using the Muse™ cell analyzer (Luminex, Austin, TX, USA).

### 4.9. Caspase 3/7 Assay

Cells were seeded, treated, incubated, and harvested as in Section 2.8. Approximately 5 µL of caspase-3/7 working reagent (Muse™ caspase-3/7 containing a DEVD peptide with a recognition site for caspase 3 and 7 conjugated to a fluorescent DNA binding dye) was added to the cell suspension (50 µL of BA assay buffer). The cells were incubated at 37 °C for 30 min, followed by the addition of 7-AAD working solution (150 µL). The suspensions were thoroughly mixed and analyzed as in Section 4.8.

### 4.10. Oxidative Stress Assay

Cells were seeded, treated, incubated, and harvested as in Section 4.8. The resuspended cells (in 10 μL of BA assay buffer) were maintained at a density of 1 × 10^7^ cells/mL. The Muse^®^ Oxidative Stress Reagent working solution (1:80 in BA assay buffer) was added to 10 µL of the cell suspension and incubated at 37 °C for 30 min, in the dark. ROS levels were assessed as in Section 4.8.

### 4.11. Apoptosis Assay

Apoptosis was determined by staining cells with the vital dual-stain system of acridine orange (AO) and ethidium bromide (EB) [39,92]. Cells were seeded at a density of 1.5 × 10^5^ cells/well in a 48-well plate containing 250 μL of growth medium and incubated at 37 °C for 24 h. Thereafter, the medium was replenished, and the cells were treated with the respective AuNCs and their nanocomplexes at their IC50 values and incubated for 48 h at 37 °C. The assay was conducted in triplicate, with untreated cells used as positive controls. Following incubation, the cells were washed with 200 μL of PBS, then stained with 10 μL of the AO/EB (1:1 *v*/*v*) solution (100 μg/mL each in PBS) with shaking for 5 min. The cells were washed with PBS (200 μL), and then viewed under an Olympus fluorescence microscope (200× magnification). Images were captured using a CC12 fluorescence camera (Olympus Co., Tokyo, Japan).

### 4.12. Statistical Analysis

Data are presented as the mean ± standard deviation (SD; *n* = 3). Data were analyzed using GraphPad Prism Version 7.3 (GraphPad Software Inc., San Diego, CA, USA). A two-way ANOVA with a post-hoc Tukey test was used to identify significant differences among the groups, while differences between two values were performed using an unpaired Student’s *t*-test, and a one-way ANOVA was used to compare test means with the control mean. Differences were considered statistically significant at * *p* < 0.05 and ** *p* < 0.01, while # denotes no statistical significance and *n* denotes the number of technical replicates (*n* = 3). All experiments were conducted in triplicate (*n* = 3).

## 5. Conclusions

This preliminary study highlights the merits of PAMAM-modified AuNCs for drug delivery and for improving drug bioavailability and pharmacokinetics. Furthermore, it provides a proof-of-concept for the subcellular delivery of therapeutics, with a measurable impact at lower doses and negligible side effects in normal cells. While targeted nanocomplexes demonstrated significant cytotoxicity in selected cancer cells, further investigation is required to conclusively determine the precise apoptotic mechanism induced and to quantify the degree of expression. Although observed patterns suggest mitochondrial involvement, this needs to be further quantified. We have provided preliminary evidence for the folate receptor-dependent uptake of FA-capped AuNCs and the possibility of TPP+ effects on mitochondrial membrane crossing. Additional mechanistic confirmations, receptor knockdown, or confocal colocalization studies may be undertaken in the future to fully confirm receptor-mediated endocytosis and TPP^+^-facilitated mitochondrial localization. Additionally, the use of non-malignant breast epithelial cells, in addition to HEK293 cells, can provide further evidence of selectivity and targeting. Further research and optimizations are, however, required to fully exploit the potential of these nano-delivery systems. In-depth studies are needed to assess their potential for clinical applications and to examine the fate of AuNCs in vitro and in vivo.

## Figures and Tables

**Table 1:** List of doxorubicin encapsulation efficiency in the different AuNCs.

Nanocomplex	µg DOX Per mg Carrier	Encapsulation Efficiency (%)
AuNC-DOX	377.6	89.5
PAM-AuNC-DOX	360.3	85.4
PEG-PAM-AuNC-DOX	338.8	80.3
FA-PAM-AuNC-DOX	352.7	83.6
FA-PEG-PAM-AuNC-DOX	334.2	79.2
TPP-PAM-AuNC-DOX	343.9	81.5
TPP-PEG-PAM-AuNC-DOX	330.8	78.4
TPP-FA-PAM-AuNC-DOX	342.6	81.2
TPP-FA-PEG-PAM-AuNC-DOX	340.1	80.6

**Table 2:** Hydrodynamic size (HS), ζ potential measurements, and polydispersity index (PDI) of FAuNCs and their drug nanocomplexes. Data are presented as the mean diameter or ζ potential ± standard deviation (*n* = 3).

Nanoclusters					Drug Nanocomplex			
NC	HS Size (nm)	EM (nm)	ζ Potential (mV)	PDI	HS Size (nm)	EM (nm)	ζ Potential (mV)	PDI
AuNC	50.2 ± 9.6	3	−38.8 ± 0.5	0.0365	-	-	-	-
PAM-AuNC	105.3 ± 3.6	5	40.6 ± 9.9	0.00117	101.3 ± 17.9	9	31.0 ± 3.2	0.0312
PEG-PAM-AuNC	113.3 ± 18.8	8	39.3 ± 5.2	0.0275	122.7 ± 11.9	10	28.9 ± 1.6	0.0094
FA-PAM-AuNC	90.2 ± 16.6	5	38.0 ± 8.5	0.0337	77.8 ± 18.5	12	27.1 ± 0.3	0.1342
FA-PEG-PAM-AuNC	101.7 ± 12.1	10	42.8 ± 15.7	0.0141	103.6 ± 0.8	12	27.9 ± 2.8	0.00006
TPP-PAM-AuNC	103.0 ± 8.1	5	43.1 ± 4.5	0.00618	145.4 ± 2.5	12	29.8 ± 3.9	0.000296
TPP-PEG-PAM-AuNC	109.1 ± 16.0	8	39.8 ± 8.5	0.0215	99.9 ± 22.4	10	29.2 ± 0.1	0.1801
TPP-FA-PAM-AuNC	118.8 ± 5.3	7	43.4 ± 5.6	0.00199	109.2 ± 21.8	11	26.3 ± 1.3	0.0398
TPP-FA-PEG-PAM-AuNC	116.0 ± 9.4	9	36.2 ± 5.4	0.00657	113.3 ± 6.4	13	32.0 ± 1.0	0.00319

**Table 3:** Correlation coefficient (R^2^) of selected kinetic models on the release profile of DOX.

	PAM-AuNC-DOX			PEG-PAM-AuNC-DOX			FA-PAM-AuNC-DOX			FA-PEG-PAM-AuNC-DOX		
pH	4.5	6.5	7.4	4.5	6.5	7.4	4.5	6.5	7.4	4.5	6.5	7.4
Zero order	0.934	0.921	0.896	0.945	0.912	0.903	0.928	0.899	0.884	0.937	0.908	0.891
First order	0.983	0.976	0.962	0.988	0.971	0.968	0.981	0.965	0.959	0.984	0.973	0.961
Higuchi	0.972	0.962	0.948	0.978	0.958	0.954	0.969	0.951	0.942	0.974	0.960	0.947
Hixon–Crowel	0.967	0.958	0.941	0.974	0.953	0.949	0.964	0.945	0.936	0.970	0.955	0.941
Korsmeyer–Peppas	0.985 (*n*) ^a^ 0.52	0.978 (*n*) ^a^ 0.58	0.965 (*n*) ^a^ 0.41	0.990 (*n*) ^a^ 0.44	0.973 (*n*) ^a^ 0.49	0.970 (*n*) ^a^ 0.39	0.983 (*n*) ^a^ 0.48	0.968 (*n*) ^a^ 0.53	0.962 (*n*) ^a^ 0.42	0.986 (*n*) ^a^ 0.46	0.975 *(n*) ^a^ 0.51	0.964 (*n*) ^a^ 0.40

^a^ (*n*): Korsmeyer–Peppas release exponent.

**Table 4:** Correlation coefficient (R^2^) of tested kinetic models on the release profile of DOX from the TPP^+^ containing AuNC nanocomplexes.

	TPP-PAM-AuNC-DOX			TPP-PEG-PAM-AuNC-DOX			TPP-FA-PAM-AuNC-DOX			TPP-FA-PEG-PAM-AuNC-DOX		
pH	4.5	6.5	7.4	4.5	6.5	7.4	4.5	6.5	7.4	4.5	6.5	7.4
Zero order	0.941	0.915	0.902	0.932	0.906	0.889	0.926	0.897	0.882	0.939	0.911	0.895
First order	0.986	0.974	0.969	0.982	0.972	0.960	0.980	0.964	0.958	0.985	0.975	0.965
Higuchi	0.976	0.961	0.955	0.971	0.958	0.945	0.968	0.950	0.941	0.975	0.961	0.951
Hixon–Crowel	0.972	0.956	0.950	0.966	0.953	0.939	0.963	0.944	0.935	0.971	0.956	0.945
Korsmeyer–Peppas	0.988 (*n*) ^a^ 0.54	0.976 (*n*) ^a^ 0.47	0.971 (*n*) ^a^ 0.38	0.984 (*n*) ^a^ 0.49	0.974 (*n*) ^a^ 0.45	0.963 (*n*) ^a^ 0.37	0.982 (*n*) ^a^ 0.51	0.967 (*n*) ^a^ 0.44	0.961 (*n*) ^a^ 0.39	0.987 (*n*) ^a^ 0.47	0.977 (*n*) ^a^ 0.43	0.968 (*n*) ^a^ 0.38

^a^ (*n*): Korsmeyer–Peppas release exponent.

**Table 5:** IC_50_ values of DOX, targeted, and untargeted nanocomplexes in vitro.

	HEK293	Caco-2	MDA-MB-231	HeLa	MCF-7
	Estimated IC_50_ Values (µg/mL)				
PAM-AuNC-DOX	236.8	112.9	86.7	74.8	87.3
PEG-PAM-AuNC-DOX	207.4	96.2	122.4	69.3	93.6
FA-PAM-AuNC-DOX	210.9	108.3	79.2	54.9	63.2
FA-PEG-PAM-AuNC-DOX	181.6	106.8	79.8	50.3	59.5
TPP-PAM-AuNC-Dox	229.1	85.3	65.4	45.6	67.8
TPP-PEG-PAM-AuNC-DOX	205.9	117.5	60.2	45.1	55.1
TPP-FA-PAM-AuNC-DOX	178.6	113.6	55.3	45.8	49.2
TPP-FA-PEG-PAM-AuNC-DOX	168.3	124.2	50.1	45.9	45.4
DOX	148.2	138.7	206.4	97.5	126.4

## Data Availability

The original contributions presented in this study are included in the article and Supplementary Materials. Further inquiries can be directed to the corresponding author.

## Supplementary Materials

The following supporting information can be downloaded at: https://www.mdpi.com/article/10.3390/ph19040572/s1. Supplementary Figure S1: NTA zeta potential and hydrodynamic size distributions for the (**A**) AuNPs and FAuNCs +DOX- (**B**) PAM-AuNC, (**C**) PEG-PAM-AuNC, (**D**) FA-PAM-AuNC, (**E**) FA-PEG-PAM-AuNC, (**F**) TPP-PAM-AuNC, (**G**) TPP-PEG- PAM-AuNC, (**H**) TPP-FA-PAM-AuNC, and (**I**) TPP-FA-PEG-PAM-AuNC. Supplementary Figure S2: Cytographs of targeted and untargeted nanocomplexes on mitochondrial membrane potential in Caco-2, MDA-MB-231, HeLa and MCF-7 cells. Control = untreated cells. (**A**) Free drug control, (**B**) PAM-AuNC-DOX treatment, (**C**) PEG-PAM-AuNC-DOX treatment, (**D**) FA-PAM-AuNC-DOX treatment, (**E**) FA-PEG-PAM-AuNC-DOX treatment, (**F**) TPP-PAM-AuNC-DOX treatment, (**G**) TPP-PEG-PAM-AuNC-DOX treatment, (**H**) TPP-FA-PAM-AuNC-DOX treatment, and (**I**) TPP-FA-PEG-PAM-AuNC-DOX treatment. Supplementary Figure S3. Cytographs of the effect of the targeted and untargeted nanocomplexes on Caspase 3/7 activity in Caco-2, MDA-MB-231, HeLa, and MCF-7 cells. Control = untreated cells. (**A**) Cells treated with free DOX, (**B**) PAM-AuNC-DOX treatment, (**C**) PEG-PAM-AuNC-DOX treatment, (**D**) FA-PAM-AuNC-DOX treatment, (**E**) FA-PEG-PAM-AuNC-DOX treatment, (**F**) TPP-PAM-AuNC-DOX treatment, (**G**) TPP-PEG-PAM-AuNC-DOX treatment, (**H**) TPP-FA-PAM-AuNC-DOX treatment, and (**I**) TPP-FA-PEG-PAM-AuNC-DOX treatment. Supplementary Figure S4. Cytographs showing the effect of the targeted and untargeted nanocomplexes on oxidative stress progression in Caco-2, MDA-MB-231, HeLa, and MCF-7 cells. Control = untreated cells. (**A**) Free DOX treatment, (**B**) PAM-AuNC-DOX treatment, (**C**) PEG-PAM-AuNC-DOX treatment, (**D**) FA-PAM-AuNC-DOX treatment, (**E**) FA-PEG-PAM-AuNC-DOX treatment, (**F**) TPP-PAM-AuNC-DOX treatment, (**G**) TPP-PEG-PAM-AuNC-DOX treatment, (**H**) TPP-FA-PAM-AuNC-DOX treatment, and (**I**) TPP-FA-PEG-PAM-AuNC-DOX treatment.

## Abbreviations

The following abbreviations are used in this manuscript:

AO	Acridine Orange
AuNC	Gold nanoclusters
Caco-2	Human colon adenocarcinoma
DMSO	Dimethyl sulfoxide
DOX	Doxorubicin
EB	Ethidium Bromide
EE	Encapsulation Efficiency
FA	Folic acid/Folate
FAuNCs	Functionalized gold nanoclusters
FBS	Fetal bovine serum
FR	Folate receptor
FTIR	Fourier transform infra-red analysis
HEK293	Human embryonic kidney
HeLa	Human cervical carcinoma
MCF-7	Human breast adenocarcinoma
MDA-MB-231	Human epithelial breast cancer—triple negative
NPs	nanoparticles
NTA	Nanoparticle tracking analysis
PAMAM	poly(amidoamine) dendrimers
SPR	Surface plasmon resonance
STPP	sodium tripolyphosphate
TEM	Transmission electron microscopy
TPP/TPP^+^	triphenylphosphonium cation
