# Supplementary material for: In Vitro Doxorubicin Delivery Using TPP–Folate-Dendrimer-Functionalized Gold Nanoclusters
Source: Pharmaceuticals (Basel). 2026 Apr 2;19(4):572. doi: 10.3390/ph19040572 (PMC13118594; doi:10.3390/ph19040572)
Supplement: Supplementary file 1 [file pharmaceuticals-19-00572-s001.zip › pharmaceuticals-4186448-supplementary.pdf]

pharmaceuticals

Supplementary Materials

In Vitro Doxorubicin Delivery Using TPP–Folate-Dendrimer-Functionalized Gold Nanoclusters

Mkhuseli Zenze and Moganavelli Singh *

Nano-Gene and Drug Delivery Group, Discipline of Biochemistry, University of KwaZulu-Natal, Private Bag X54001, Durban 4001, South Africa; 213515339@stu.ukzn.ac.za
* Correspondence: singhm1@ukzn.ac.za; Tel.: +27-31-2607170

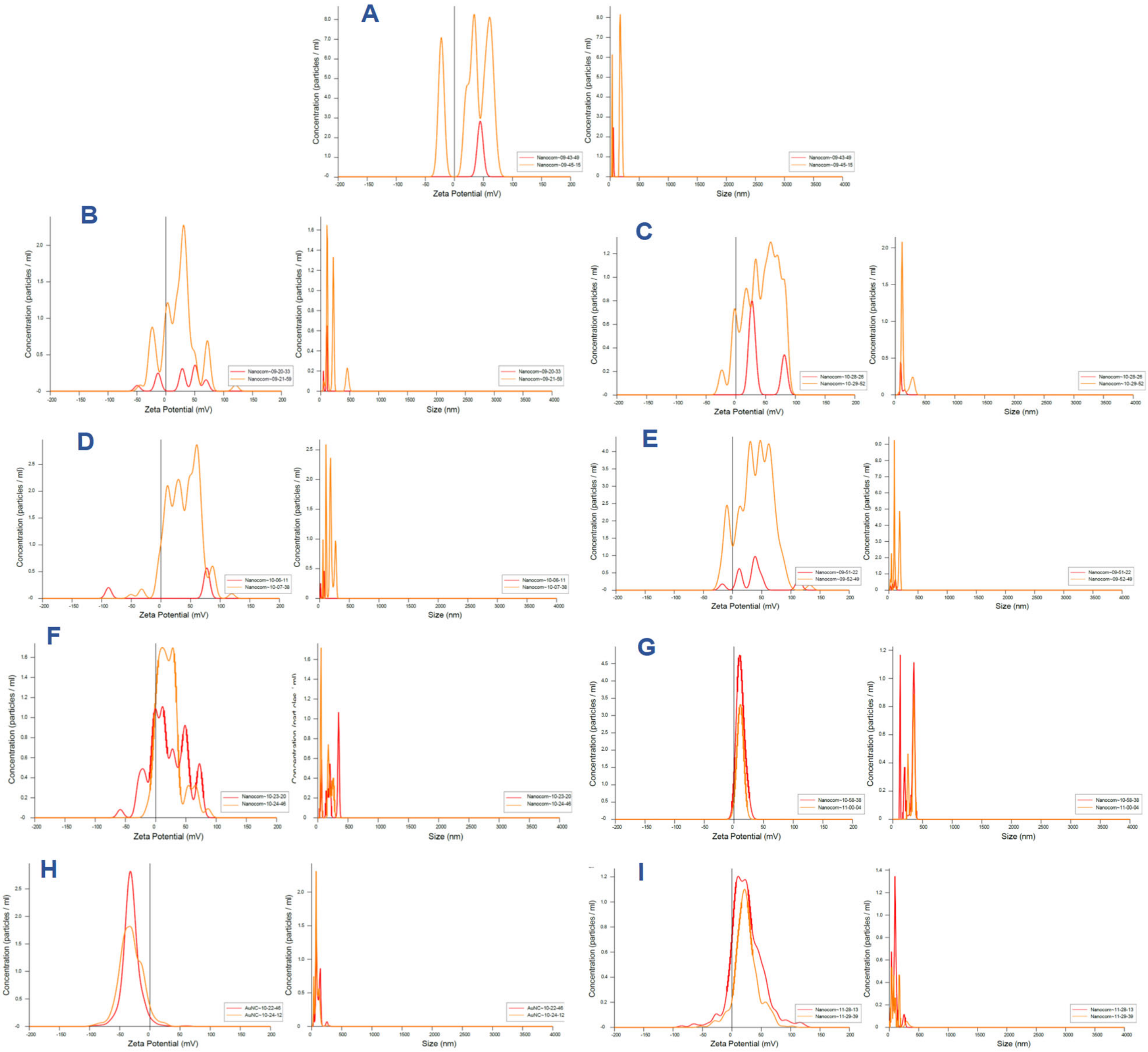

Figure S1. NTA Zeta potential and hydrodynamic size distributions for (A) AuNPs and FAuNCs +DOX- (B) PAM-AuNC, (C) PEG-PAM-AuNC, (D) FA-PAM-AuNC, (E) FA-PEG-PAM-AuNC, (F) TPP-PAM-AuNC , (G) TPP-PEG- PAM-AuNC, (H) TPP-FA-PAM-AuNC, and (I) TPP-FA-PEG-PAM-AuNC.

 https://doi.org/10.3390/ph19040572

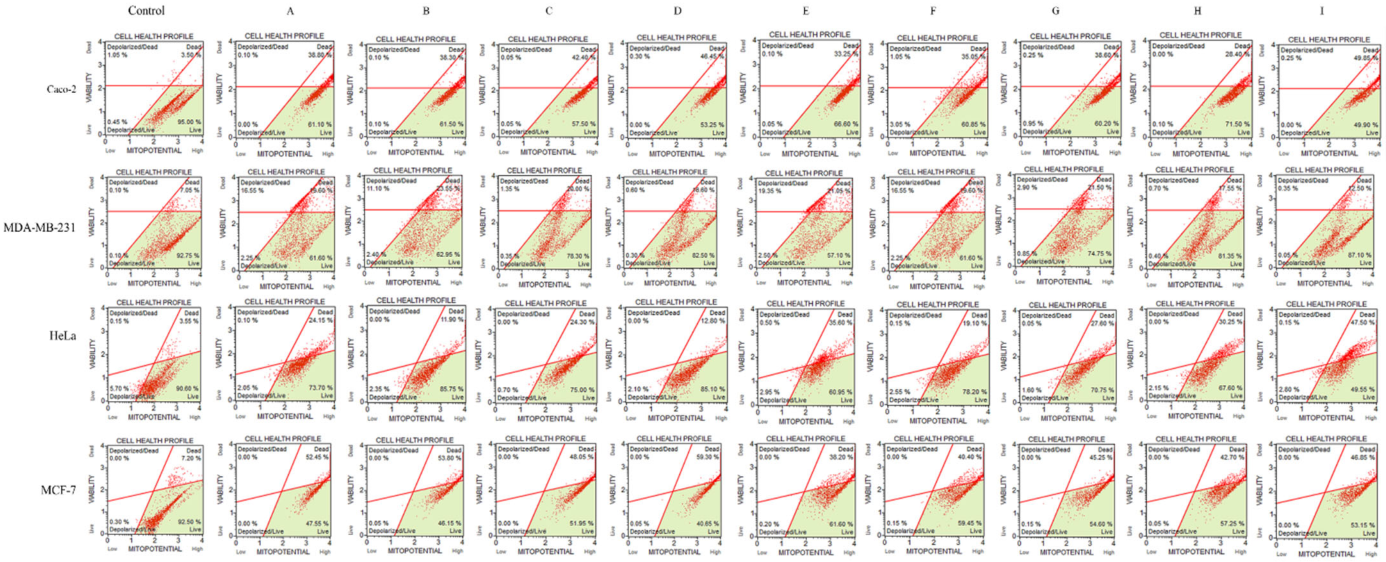

Figure S2. Cytographs of targeted and untargeted nanocomplexes on mitochondrial membrane potential in Caco-2, MDA-MB-231, HeLa and MCF-7 cells. Control = untreated cells, (A) Free drug control, (B) PAM-AuNC-DOX treatment, (C) PEG-PAM-AuNC-DOX treatment, (D) FA-PAM-AuNC-DOX treatment, (E) FA-PEG-PAM-AuNC-DOX treatment, (F) TPP-PAM-AuNC-DOX treatment, (G) TPP-PEG-PAM-AuNC-DOX treatment, (H) TPP-FA-PAM-AuNC-DOX treatment, and (I) TPP-FA-PEG-PAM-AuNC-DOX treatment.

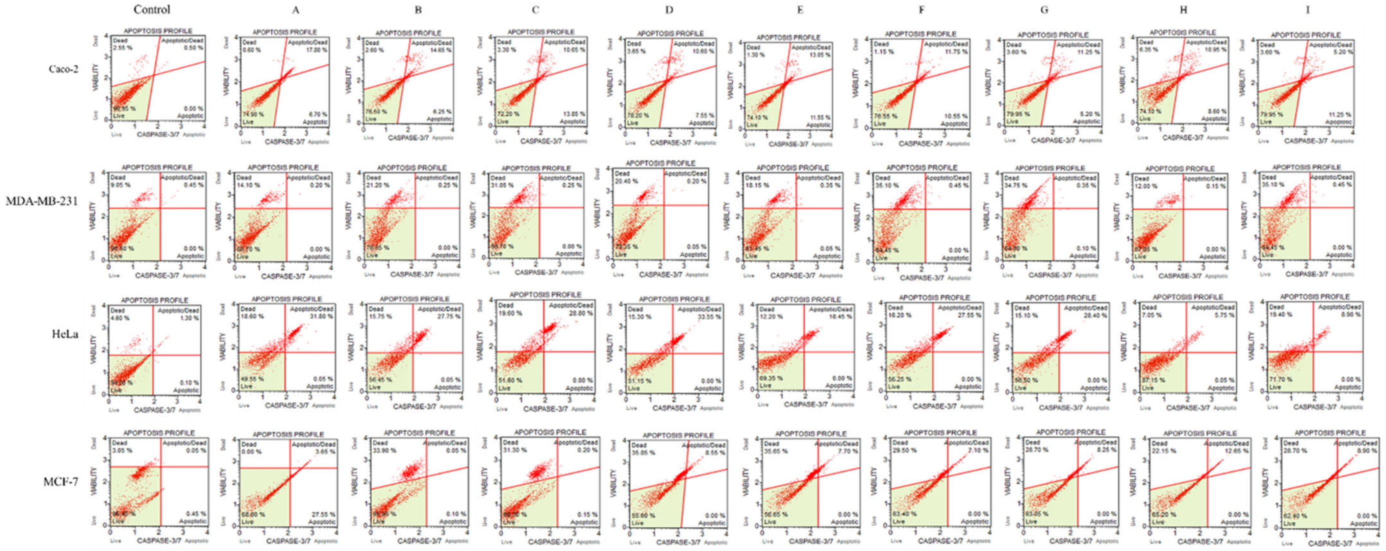

Figure S3. Cytographs of the effect of the targeted and untargeted nanocomplexes on Caspase 3/7 activity in Caco-2, MDA-MB-231, HeLa and MCF-7 cells. Control =untreated cells, (A) Cell treated with free DOX, (B) PAM-AuNC-DOX treatment, (C) PEG-PAM-AuNC-DOX treatment,(D) FA-PAM-AuNC-DOX treatment, (E) FA-PEG-PAM-AuNC-DOX treatment, (F) TPP-PAM-AuNC-DOX treatment, (G) TPP-PEG-PAM-AuNC-DOX treatment, (H) TPP-FA-PAM-AuNC-DOX treatment, and (I) TPP-FA-PEG-PAM-AuNC-DOX treatment.

https://doi.org/10.3390/ph19040572

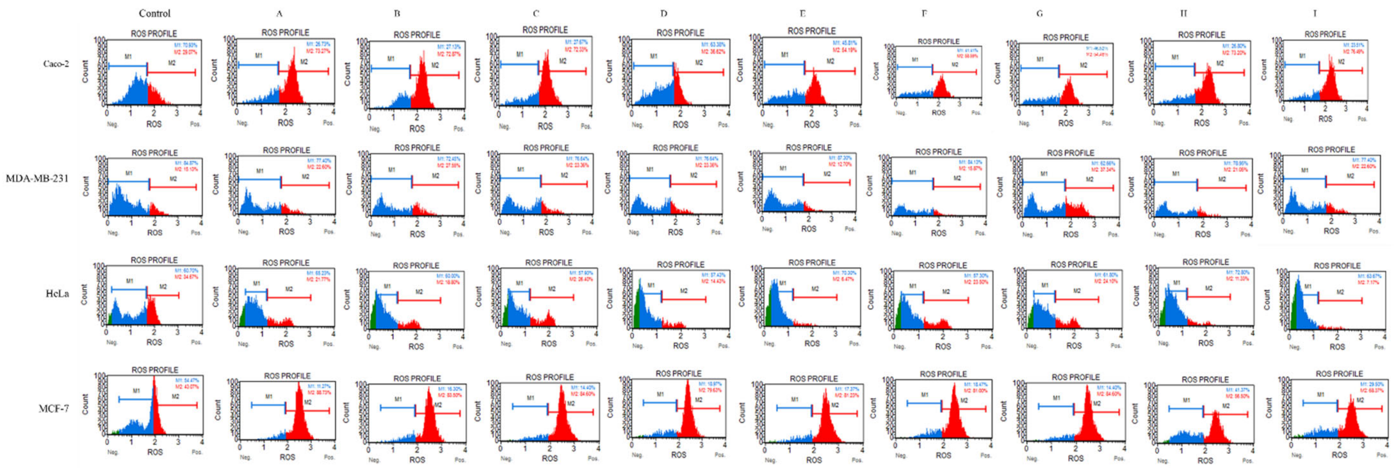

Figure S4. Cytographs showing the effect of the targeted and untargeted nanocomplexes on oxidative stress progression in Caco-2, MDA-MB-231, HeLa and MCF-7 cells. Control =untreated cells, (A) free DOX treatment, (B) PAM-AuNC-DOX treatment, (C) PEG-PAM-AuNC-DOX treatment, (D) FA-PAM-AuNC-DOX treatment, (E) FA-PEG-PAM-AuNC-DOX treatment, (F) TPP-PAM-AuNC-DOX treatment, (G) TPP-PEG-PAM-AuNC-DOX treatment, (H) TPP-FA-PAM-AuNC-DOX treatment, and (I) TPP-FA-PEG-PAM-AuNC-DOX treatment.

https://doi.org/10.3390/ph19040572
